# Fault-Diagnosis-Aware Adaptive Policy Switching for Robust Map-Free Mobile Robot Navigation Under Sensor and Control Degradation

**DOI:** 10.3390/s26206452

**Published:** 2026-10-12

**Authors:** Taner Yilmaz, Omur Aydogmus

**Affiliations:** Faculty of Technology, Department of Mechatronics Engineering, Fırat University, Elazig 23119, Türkiye; oaydogmus@firat.edu.tr

**Keywords:** mobile-robot navigation, fault diagnosis, policy switching, proximal policy optimization, LiDAR degradation, fault-tolerant control, reinforcement learning, adaptive control

## Abstract

**Highlights:**

**What are the main findings?**

- Explicit temporal fault diagnosis enabled online routing among six fault-specialized navigation policies.
- Diagnosed switching improved dynamic-transition success over robust PPO in all three policy-training seeds.

**What are the implications of the main findings?**

- Mechanism controls linked performance to fault-specific specialist competence and the fault-to-specialist correspondence.

**Abstract:**

Heterogeneous sensor and control degradation can destabilize direct sensor-to-action navigation. This study presents a fault-diagnosis-aware adaptive policy-switching framework that combines a calibrated seven-class temporal health-state classifier with hysteretic routing among nominal, robust-fallback, and six fault-specialized proximal policy optimization (PPO) policies. Unlike monolithic robust reinforcement learning, the architecture makes the inferred degradation mode an explicit and auditable control variable and separates health-state inference from behavior selection. Across a large held-out simulation evaluation with three independently trained policy seeds, diagnosed switching improved the central severity-0.55 static benchmark and within-episode transition outcomes relative to robust PPO, while the broader severity sweep showed regime-dependent behavior. Diagnosed switching also retained an aggregate advantage under interaction-matched robust training. Mechanism controls showed that the gain depended on fault-specific specialist competence and correct fault-to-specialist correspondence rather than policy-bank capacity alone. Single-thread inference remained well below the 200 ms control interval. Within the evaluated simulation scope, the results support explicit online diagnosis and behaviorally matched policy selection as a practical strategy for robust map-free navigation under heterogeneous degradation.

## 1. Introduction

Map-free mobile-robot navigation maps local sensory observations and relative goal information directly to motion commands without requiring a prebuilt global obstacle map. Recent LiDAR-based, multimodal, and memory-augmented deep reinforcement learning (DRL) systems have demonstrated strong navigation performance in unknown environments [1,2,3], while recent reviews continue to identify generalization, dynamic-obstacle handling, sensor reliability, and sim-to-real deployment as persistent challenges [4]. PPO remains a practical continuous-control baseline because its clipped objective supports stable repeated updates [5]. However, direct sensor-to-action coupling also creates a short failure path: corrupted ranges, stale observations, or delayed actuation can immediately alter commanded motion.

Current approaches address this reliability problem from several complementary directions. Monolithic robust reinforcement learning uses randomization or temporal context so that one policy can tolerate a distribution of disturbances [6,7,8]; its strength is architectural simplicity, but heterogeneous faults with different control implications must share one policy capacity. Fault detection, isolation, and reconfiguration (FDIR) instead separates health-state inference from accommodation [9,10]; this provides interpretable fault information, although the downstream recovery action is commonly an estimator, conventional controller, or system reconfiguration rather than learned navigation-policy selection. Modular and mixture-of-experts control provides specialization through gating [11,12], but many learned systems rely on latent adaptation or failure information rather than an explicit calibrated diagnosis obtained online from degraded navigation signals. These complementary strengths and limitations motivate the specific gap considered here: explicit online diagnosis coupled to behaviorally matched policy selection for LiDAR-based wheeled map-free navigation under heterogeneous sensing and control degradation.

**Figure 1:**
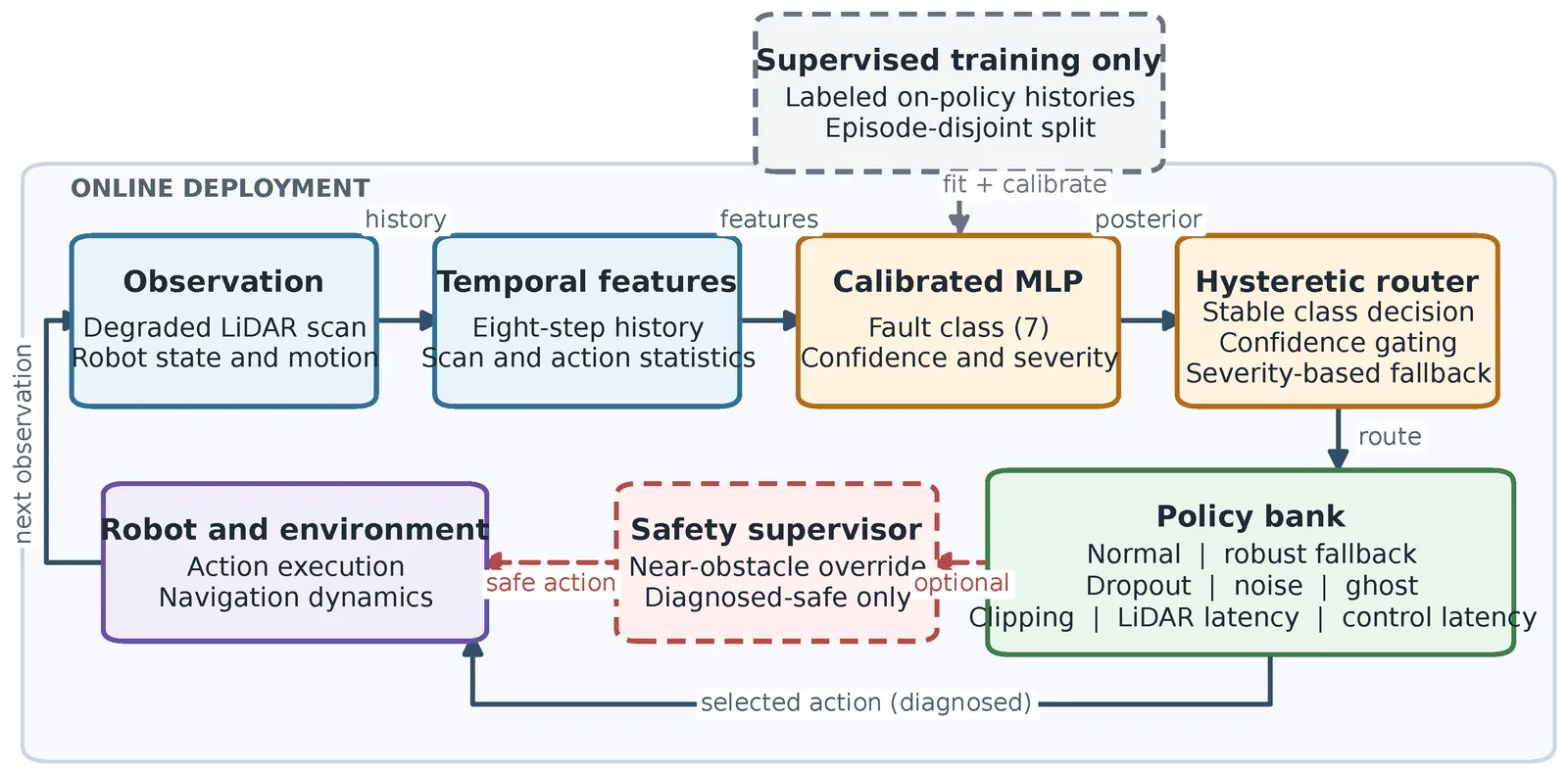
Fault-diagnosis-aware adaptive policy-switching architecture. Recent degraded light detection and ranging (LiDAR) measurements, requested actions, and observed motion were converted to temporal features. The calibrated multilayer perceptron (MLP) diagnoser estimated fault class, confidence, and severity. A hysteretic router selected the nominal, robust-fallback, or one of six specialist policies. The safety supervisor was enabled only for the diagnosed-safe method. Ground-truth fault labels were used for supervised diagnosis training and evaluation annotations but were unavailable to the deployed controller.

The objective of this study was therefore to determine whether an explicit, calibrated health-state estimate can support stable within-episode switching among fault-specialized navigation policies and preserve a measurable advantage over monolithic robust PPO when the degradation mode changes during operation. The architecture shown in Figure 1 uses a temporal classifier over recent LiDAR, requested-action, and observed-motion histories to predict one of seven health states together with confidence and severity. A hysteretic router then selects the nominal policy, a robust fallback, or one of six PPO specialists for LiDAR dropout, noise, ghost returns, range clipping, LiDAR latency, and control latency. Temperature scaling was used because routing explicitly depends on confidence [13]. An optional safety supervisor was evaluated separately so that the effect of diagnosis-aware routing was not conflated with conservative action intervention.

The evaluation used three independently trained policy seeds and a broad held-out simulation protocol spanning static faults, within-episode transitions, interaction-matched robust training, distribution-shift conditions, mechanism controls, and computational profiling. The added value relative to a monolithic robustness formulation is an auditable diagnosis–decision interface in which the health estimate, route acquisition, specialist correspondence, and computational consequences can be examined separately rather than absorbed into one universal policy. The formal contribution statement is given after the critical literature synthesis in Section 2.5. The remainder of the paper is organized as follows. Section 2 critically reviews map-free reinforcement learning, robust navigation, fault diagnosis and fault-tolerant control, and modular online adaptation, and then positions the present contribution against these strands. Section 3 describes the simulation environment, degradation models, policy training, diagnosis-and-routing framework, evaluation protocol, and statistical analysis. Section 4 reports diagnosis, navigation, transition, mechanism, robustness, and computational results. Section 5 discusses interpretation and limitations, and Section 6 concludes the study and outlines future work.

## 2. Related Work

The literature is reviewed below through four complementary strands: map-free DRL navigation, robustness to sensor corruption and delay, explicit fault diagnosis and fault-tolerant control, and online adaptation or modular policy selection. The purpose is not only to enumerate prior methods but to distinguish what each strand handles well and what remains unresolved for the present problem. In particular, the comparison focuses on whether the health state is inferred explicitly, whether recovery changes the learned navigation behavior, and whether adaptation is evaluated during within-episode fault transitions rather than only under stationary degradation.

### 2.1. Map-Free Reinforcement Learning Navigation

Recent journal literature has demonstrated several complementary directions in learning-based mobile-robot navigation. LiDAR observations have been used directly with continuous-action deep reinforcement learning [1]; multimodal systems have combined LiDAR, camera, odometry, and goal information [2]; and hierarchical reinforcement learning with memory has been investigated for map-free navigation in unknown environments [3]. Recent surveys cover value-based, policy-based, recurrent, multimodal, and hybrid navigation approaches while emphasizing generalization, dynamic environments, and real-world deployment as continuing challenges [4]. A recent LiDAR-only benchmark compared major on- and off-policy deep reinforcement learning algorithms under a common map-free navigation protocol and examined zero-shot transfer to ROS/Gazebo and TurtleBot3 hardware [14]. PPO provides a stable and implementation-friendly policy-gradient baseline for continuous control [5]. Complementary robotics research has also examined exploration–exploitation trade-offs and uncertainty-aware path optimization in complex environments [15]. In contrast to work primarily targeting nominal navigation quality, environmental generalization, reward design, or sim-to-real transfer, the present study held the navigation task fixed and isolated degradation in the observation and control channels.

### 2.2. Robust Navigation Under Sensor Corruption and Delay

Domain randomization aims to learn a policy that performs well over a distribution of simulator parameters rather than a single nominal model. Recent reviews cover randomization of observation, action, dynamics, geometry, and other uncertain quantities [6], while offline approaches can estimate a randomization distribution from target-domain trajectories [7]. In mobile navigation, multimodal deep reinforcement learning has fused LiDAR, camera, odometry, and goal information [2]; learned gating has been used to weight camera and LiDAR features according to sensor availability [16]; and fault-tolerant map-free policies have been trained end to end for broken range sensors [17]. The robust-PPO baseline followed this monolithic robustness paradigm; the present study instead examined explicit online fault diagnosis and within-episode routing among fault-specialized policies.

Observation and control delays deserve separate treatment from independent sensor noise. A delayed scan may remain physically plausible while describing an earlier scene, and a delayed command changes the action that actually generated the current motion. Delay-aware reinforcement learning formalizes this loss of temporal alignment and commonly restores a Markov representation by augmenting the state or model with past actions and observations [8]. The study did not propose a general delay-aware RL algorithm; instead, it treated LiDAR and control latency as diagnosable health modes and evaluated whether specialist routing provided a practical alternative to forcing one policy to absorb both temporal and amplitude degradations.

### 2.3. Fault Diagnosis and Fault-Tolerant Control

Classical FDIR pipelines distinguish detection and isolation from subsequent fault accommodation or control reconfiguration [9]. More recent FDD reviews emphasize the continuing use of model-based residuals together with intelligent and data-driven diagnosis [10]. A recent state-of-the-art review of UAV fault detection and diagnosis similarly emphasizes the growing role of intelligent, data-driven, and real-time diagnostic methods while cataloguing their respective strengths and limitations [18]; its focus is aerial-platform health monitoring rather than diagnosis-conditioned selection among learned map-free navigation policies. For mobile robots, recent studies include data-driven fault diagnosis from heterogeneous sensor streams [19], fault-tolerant localization through multiple-model Kalman-filter sensor fusion [20], Bayesian sensory anomaly detection and recovery [21], proactive navigation-failure detection with multi-sensor fusion [22], and LiDAR fault isolation followed by decentralized formation reconfiguration [23]. Most of these methods assume a defined fault or anomaly model and associate detection or diagnosis with an estimator, controller, or recovery action. By contrast, broader anomaly-detection research in autonomous robotic missions also considers deviations that need not belong to a predefined fault class and emphasizes temporal as well as spatiotemporal evidence [24]. The diagnoser in the present study was deliberately closed-set and classified nominal operation plus six injected modes; unknown-fault rejection is therefore outside the current claims. The contribution is the downstream connection from calibrated health-state inference to learned navigation-policy selection and the explicit measurement of behavior during onset, recovery, and cross-fault transitions.

### 2.4. Online Adaptation and Modular Policies

Mixture-of-experts architectures provide a general mechanism for dividing a complex input space among specialists through a learned gating function [11]. More recent modular deep reinforcement learning frameworks have separated navigation, training, and learning components to support flexible autonomous-navigation pipelines [25]. A related adaptive-control line jointly learns a control policy and a state estimator, using temporal sensor information to support robust locomotion under changing operating conditions [26]. Recurrent integration of exteroceptive and proprioceptive information has also enabled robust physical locomotion [27], while model-free reinforcement learning has been used explicitly for failure recovery and fault-tolerant locomotion [28].

A recent preprint further proposed fault-aware mixture-of-experts control in which actuator-failure information activates specialized quadrupedal locomotion experts [12]. Accordingly, the present work does not claim to be the first modular or fault-aware RL system. Its distinction lies in four coupled design choices: the robot is a differential-drive platform performing LiDAR-based map-free navigation; the health state is inferred from degraded LiDAR, requested actions, and observed motion; the router makes confidence- and severity-aware hard selections among nominal, robust, and six specialist policies; and evaluation emphasizes within-episode fault onset, recovery, cross-fault switching, and the delay between a physical transition and an operationally correct route.

### 2.5. Positioning of the Present Work

Taken together, the literature suggests four broad strategies: monolithic robustness through randomized or history-aware training, explicit FDD followed by conventional reconfiguration, latent online adaptation of a universal policy, and modular expert selection. Their strengths are complementary, but none directly resolves all three requirements examined here: explicit diagnosis from degraded navigation signals, learned fault-matched behavior selection, and measured adaptation during onset, recovery, and cross-fault transitions. The developed system connects these strands using an explicit, calibrated diagnosis as the routing variable for a bank of map-free navigation policies. The central empirical question is therefore not only whether specialists outperform a monolithic robust policy under stationary faults, but whether diagnosis and routing remain sufficiently fast and stable to preserve that advantage when the degradation mode changes within an episode. This synthesis isolates the specific niche of the proposed framework and avoids treating map-free navigation, fault diagnosis, and modular control as independent literature lists.

Against this literature gap, the principal contributions are:

1. An explicit diagnosis-to-control architecture that infers a calibrated seven-class health state online from degraded navigation signals and uses that estimate to route among nominal, robust-fallback, and fault-specialized PPO behaviors without access to the simulator fault label.
2. A hysteretic fault-accommodation mechanism that separates health-state inference from behavior selection, making confidence, severity, route acquisition, and fault-to-specialist correspondence individually auditable rather than embedding adaptation inside a single monolithic policy.
3. A transition-centered validation framework that evaluates not only stationary fault outcomes but also diagnosis delay, route delay, acquisition coverage, onset, recovery, cross-fault changes, and mechanism controls that test whether performance depends on behaviorally appropriate specialist selection.
4. A resource-aware comparison against robust, temporal, oracle, safety-supervised, and interaction-matched controls, together with explicit separation of stored policy-bank capacity from active per-step inference cost and bounded interpretation across three independent policy-training seeds.

## 3. Materials and Methods

This section describes the complete experimental pipeline used to evaluate diagnosis-aware policy switching. It first defines the navigation environment and atomic degradation models, then details policy training, temporal fault diagnosis, adaptive routing and safety controls, and finally specifies the held-out evaluation suites, statistical analysis, and reproducibility procedures. The organization follows the online information flow from degraded sensing to diagnosis, policy selection, and outcome measurement.

### 3.1. Navigation Environment

The differential-drive robot operated in a square workspace with side length 10m. The navigation simulator, robot kinematics, and base map-free observation and action design followed the experimental platform established in a previous deep reinforcement learning benchmark [14]. The present study extended that platform with explicit sensor and control fault injection, temporal fault diagnosis, fault-specialized policies, adaptive routing, and within-episode fault-transition evaluation; the diagnosis-and-routing experiments reported here were new. Eight internal rectangular walls, the start pose, and the target were sampled procedurally. Complete scene-generation ranges, placement constraints, collision logic, start/goal constraints, and ray-casting parameters are reported in Supplementary Table S1.

The robot had a wheel diameter of 66 mm and an axle length of 160 mm. At each 0.2 s step, both components of $a_{t}=\left(a_{t}^{v},a_{t}^{\omega }\right)$ were bounded to [−1,1] and converted as follows:

(1) $$\begin{array}{cc} v_{t} & =0.11(a_{t}^{v}+1),\\ \omega_{t} & =2.84a_{t}^{\omega }. \end{array}$$

Thus, $v_{t}\in \left[0,0.22\right]$ m/s and $\omega_{t}\in \left[-2.84,2.84\right]$ rad/s. The factor 0.11 is one half of the permitted 0.22 m/s forward-speed span, so the affine mapping converts the normalized action interval [−1,1] exactly to [0,0.22] m/s; the factor 2.84 maps the normalized angular action directly to the platform limit of ±2.84 rad/s. These command limits were inherited from the previously validated simulation platform and were held fixed across all methods; the reported wheel diameter and axle length define the differential-drive geometry but were not used to retune the software action limits in this study. The 126-dimensional policy observation contained the sine and cosine of target bearing, normalized linear and angular velocity, normalized target distance, recent distance change, and 120 LiDAR ranges. Rays covered $360^{\circ }$ from the robot center over [0.16,1.00] m.

An episode was considered successful when the target distance fell below 0.5 m, terminated in collision when simulator-ground-truth LiDAR clearance fell below 0.17 m, and timed out after 1000 steps. Ground-truth clearance was used for collision termination, outcome measurement, and the danger-shaping reward term; the policy and sensor-based safeguards received only the degraded scan. Thus, ground-truth clearance did not enter the controller observation or safeguards, while sensor corruption could not change the collision definition. The reward was +1 for reaching the target and −1 for collision, together with fixed progress, time, angular-motion, linear-motion, heading, danger, action-smoothness, timeout, and terminal-efficiency terms. The base reward followed the previously reported platform [14]; every term, coefficient, clipping range, and PPO hyperparameter is reproduced in Supplementary Table S2. Because all methods used the same environment and reward, comparisons isolated policy training and routing.

### 3.2. Fault Models

Let $r_{t,i}\in \left[r_{\min},r_{\max}\right]$ be clean beam *i*, $R=r_{\max}-r_{\min}$, and s∈[0,1] the normalized fault severity. Six atomic degradations were applied with explicit labels:

1. **Dropout:** each beam was replaced by $r_{\max}$ with probability 0.05+0.70s.
2. **Noise:** independent Gaussian noise $N(0,{\left(0.20sR\right)}^{2})$ was added, and the result was clipped to the valid range.
3. **Ghost returns:** with probability 0.05+0.55s, a beam was replaced by a uniformly sampled near return in $\left[r_{\min},r_{\min}+0.30R\right]$.
4. **Range clipping:** ranges were capped at $r_{\max}-0.65sR$.
5. **LiDAR latency:** the scan from d=max(1,round(1+7s)) steps earlier was returned.
6. **Control latency:** the action requested *d* steps earlier was applied while the current LiDAR scan remained unchanged.

At episode reset, the LiDAR- and action-delay histories were cleared. During the initial *d* steps, delayed LiDAR returned the earliest available valid scan and delayed control returned the earliest requested action; the same deterministic initialization rule was used in training and evaluation. The final output of every range corruption was clipped to the physical interval. A deterministic random generator was maintained independently for each environment worker. Evaluation used paired environment and fault seeds so that all methods face identical maps and stochastic corruptions within a policy seed.

### 3.3. Policy Training

Five controller roles were defined. *Normal PPO* was trained under nominal sensing. *Robust PPO* was a monolithic baseline trained with one episode-level mode sampled uniformly from nominal operation and six faults; nonzero severity was sampled uniformly from 0.15 to 0.75. *Oracle* selected the nominal or fault-matched specialist policy from the ground-truth fault label and served as a ground-truth routing reference for the fixed policy bank. It was not assumed to be a performance upper bound across all controllers or severities. At every control step, oracle routing directly selected the policy associated with the current ground-truth fault label, bypassing learned diagnosis, confidence and severity estimation, confirmation, and dwell logic. *Diagnosed* used learned diagnosis and hysteretic routing. *Diagnosed-safe* applied an additional safety supervisor. All policy-training episodes used a stationary fault mode; within-episode fault transitions were reserved for evaluation.

For each seed in {42,43,44}, nominal and robust policies were trained from scratch for 100,000 episodes. Each of the six specialists was initialized from the corresponding robust checkpoint and fine-tuned for 100,000 episodes under one fault at severity 0.55. This design isolated fault identity during specialization; generalization was subsequently evaluated at severities 0.15, 0.35, and 0.75. The resulting bank comprised 24 policy runs and 2.4 million training episodes. Training used 16 subprocess environments. PPO actor widths were 256–256–128 and critic widths were 128–128. Common settings included rollout length 1024, batch size 256, discount γ=0.9903838, and generalized advantage parameter λ=0.9851848. Nominal and robust training used a learning rate of $5.129\times 10^{-4}$, clip range 0.225, and 10 epochs. Specialist fine-tuning used a learning rate of $10^{-4}$, clip range 0.12, five epochs, and target KL 0.03.

Two monolithic temporal-budget controls were trained for each seed. *Robust-200k* continued the corresponding robust-100k checkpoint to 200,000 cumulative episodes. *Temporal-robust-200k* was trained from scratch for 200,000 episodes with the same randomization and a flattened history of eight observations. The temporal model used the same PPO hidden architecture and hyperparameters with the enlarged input. These six checkpoints tested longer monolithic optimization and direct temporal context; the temporal MLP was not a recurrent-policy baseline. All policy evaluations used the final checkpoint at the specified training budget; no test-performance-based checkpoint selection was performed.

Interaction matching was performed separately because the eight-policy bank required more environment interaction than a single robust policy. For each seed, robust-200k was continued under its unchanged randomization and PPO hyperparameters until its cumulative timestep count matched the audited total used by the corresponding policy bank and diagnoser. Targets were 153,688,672, 137,150,288, and 118,196,080 steps for seeds 42, 43, and 44. Final counts exceeded the targets by less than one 16,384-step rollout. This control matched environment interaction but not stored model capacity, optimization topology, or wall-clock cost. For each seed, the matched target summed nominal-policy training, shared robust pretraining counted once, the six incremental specialist fine-tuning trajectories, and diagnosis-data collection. The corresponding training and capacity audit is summarized in Table 1.

The policy bank used 136.3±17.8 million audited environment steps across seeds and stored eight PPO policies plus the diagnoser (1,316,416 parameters). A monolithic robust PPO stored 164,357 parameters. Only the selected PPO and diagnoser were evaluated at a control step (165,917 active parameters), so stored capacity and active inference capacity represented distinct resource measures. Thus, specialization increases the memory needed to retain the policy bank, but it does not require evaluating all specialists at each control step: online computation consists of one small diagnoser plus the single selected PPO policy. Complete historical wall-clock and peak-memory traces were not consistently available across distributed training machines.

### 3.4. Temporal Fault Diagnosis

On-policy diagnosis data were collected separately for each seed using its robust policy. The seven classes comprised nominal operation and the six degradations. Each diagnosis-data episode retained one fixed class and severity throughout; within-episode transitions were not included in classifier training. Each class contained 100 episodes; faulty severities were sampled from 0.15 to 0.75. A history length of eight and a sample stride of two were used. Episode identifiers, rather than individual windows, were split per class into approximately 65% training, 15% validation, and 20% test partitions. Consequently, temporally adjacent windows from an episode could not appear in different partitions.

The temporal feature vector contained current scan mean, standard deviation, minimum and maximum; fractions near the maximum, minimum, and either saturation boundary; mean absolute temporal range change; fraction of large temporal jumps; scan-to-scan correlation; mean requested linear action; mean absolute angular action; observed normalized linear and angular motion; and absolute requested–observed action mismatches. These 16 low-dimensional statistics retain diagnostic cues while keeping the online classifier small and auditable. Near-maximum and near-minimum fractions used 1% of the sensor span, saturation used normalized limits 0.05 and 0.95, and a large temporal jump exceeded 20% of the span. These fixed thresholds are listed in Supplementary Table S4. The severity-regression output was clipped to [0,1] before routing.

The classifier was a one-hidden-layer multilayer perceptron with 64 hyperbolic-tangent units and a seven-way softmax output. Features were standardized using training statistics. Class-balanced cross-entropy was optimized for 250 epochs with Adam, batch size 256, learning rate $10^{-3}$, and $L_{2}$ coefficient $10^{-4}$. A linear least-squares head estimated severity from the standardized features. Nominal windows were assigned a severity target of zero, whereas faulty windows retained their episode-level injected severity value. The least-squares fit used all training windows. Temperature *T* was selected on the validation episodes from 81 logarithmically spaced candidates in [0.25,4] by minimum negative log-likelihood, and the calibrated inference probabilities were

(2) $$p\left(y=k|x\right)=\frac{\exp(z_{k}/T)}{\sum_{j}\exp\left(z_{j}/T\right)}.$$

Temperature selection optimized validation negative log-likelihood rather than test performance. Values T>1 soften an overconfident softmax distribution, whereas T<1 sharpens an underconfident distribution. Minimizing validation negative log-likelihood penalizes both confidently incorrect predictions and unnecessarily diffuse probability assignments, providing a data-driven calibration trade-off without explicitly optimizing entropy or forcing an entropy-collapse criterion. Held-out accuracy, macro-F1, expected calibration error (ECE) using ten equal-width confidence bins over [0,1], and severity mean absolute error (MAE) were reported. Severity MAE was computed over the complete episode-disjoint test set, including nominal windows.

### 3.5. Adaptive Routing and Safety Ablation

Diagnosis and routing were executed online during every evaluation episode. At each 0.2 s control step, temporal features were formed from the most recent observation–action–motion history, the diagnoser produced class ${\hat{m}}_{t}$, confidence $c_{t}$, and severity ${\hat{s}}_{t}$, and the router selected the control policy for that step. Only diagnoser training, temperature selection on validation episodes, and post hoc metric aggregation were performed offline. Detection therefore did not trigger a maintenance action or physical sensor repair in the present study; it triggered control-level fault accommodation by changing the active navigation policy. The router first evaluated the severity output. If ${\hat{s}}_{t}\geq 0.90$, the robust fallback was executed immediately, independently of predicted class and confidence. The severity fallback bypassed the three-step confirmation and five-step dwell requirements; these hysteresis rules were applied only to class-based route requests. Otherwise, predictions with $c_{t}<0.55$ requested the normal policy, while confidence-qualified predictions requested the corresponding nominal or fault-specialized policy. For class-based route requests, a requested route had to persist for three consecutive steps, and the active route had to satisfy a five-step minimum dwell before a route change was accepted. Accordingly, a confidence-qualified fault diagnosis requested the corresponding fault-specialized policy, a low-confidence diagnosis requested the normal policy, and only the severity guard (${\hat{s}}_{t}\geq 0.90$) invoked the robust fallback immediately. This policy reconfiguration is the intervention strategy evaluated here; predictive maintenance, component replacement, and repair scheduling are outside the scope of the study.

The confidence threshold, confirmation requirement, dwell time, and severity-fallback threshold were fixed engineering settings applied unchanged across all reported evaluation suites and were not selected from held-out test outcomes. Because faulty severity targets used for training were limited to [0.15,0.75], the 0.90 threshold served as a conservative safeguard on the clipped regression output rather than an in-range calibrated severity threshold. Its contribution was examined through the no-fallback ablation.

The diagnosed-safe method applied a separate supervisor after routing. The supervisor first imposed an emergency stop when the observed minimum range was at most 0.18 m, except under a confidence-qualified ghost diagnosis (${\hat{m}}_{t}=\mathrm{ghost}$ and $c_{t}\geq 0.55$). Otherwise, a confidence-qualified diagnosis with severity at least 0.90 commanded zero forward velocity and clipped normalized angular action to [−0.35,0.35], equivalent to [−0.994,0.994] rad/s; severity of at least 0.65 scaled forward speed by 0.55. Five component ablations varied hysteresis, fallback, temperature calibration, and confidence thresholds.

Two mechanism-focused controls were evaluated. First, a deterministic label-derangement negative control applied the fixed cyclic fault-label mapping reported in Supplementary Table S5 to the six non-nominal diagnosis labels before routing. The normal label remained unchanged. The confidence and severity estimators, confirmation and dwell rules, and all router hyperparameters were unchanged. Only the non-nominal fault-to-specialist correspondence was altered. The two routing conditions were executed as separate closed-loop rollouts; because different specialist actions could alter subsequent trajectories and diagnostic inputs, identical diagnosis and switching sequences were not assumed. Consequently, the routing mechanism was retained while the fault-to-specialist correspondence was deliberately disrupted. The mapping is reported in Supplementary Table S5. The control was evaluated on a dedicated severity-0.55, step-50 dynamic block containing the same 18 onset, recovery, and cross-fault scenarios as the central transition suite. Each routing condition used 100 episodes per scenario and policy seed, corresponding to 1800 episodes per method and seed, 5400 episodes per method across the three seeds, and 10,800 episode records in total. Standard and label-deranged routing used identical environment and fault seeds within every matched episode. This pairing controlled environment and fault randomness but did not impose identical robot trajectories. This mechanism-test block used evaluation-seed base 71,000 and was distinct from the central dynamic benchmark, which used 200 episodes per scenario and evaluation-seed base 40,000. Second, executed policy routes were recorded at every control step in a dedicated route-trace evaluation executed separately from the mixed/unknown outcome suite. The same seven mixed and unknown condition definitions were evaluated at severities 0.35 and 0.55 for the diagnosed controller, with 100 episodes per condition and policy seed, yielding 4200 episode records. This block used evaluation-seed base 70,000 and did not reuse rollouts from the mixed/unknown outcome evaluation. Together with the full fault-policy cross-performance matrix, these controls probed fault-specific specialist competence and the closed-loop consequences of disrupting the fault-to-specialist correspondence.

### 3.6. Evaluation Protocol

The 550,500 held-out records were organized into three connected evaluation layers: (i) atomic stationary-fault suites quantified baseline competence and severity dependence; (ii) within-episode transition suites quantified onset, recovery, cross-fault adaptation, and transition-time sensitivity; and (iii) stress and mechanism suites tested mixed/unknown/correlated degradation, interaction-matched training, specialist specificity, route traces, and router ablations. Supplementary Table S6 provides the exact non-overlapping episode accounting for these layers. Evaluation comprised complementary held-out suites. The core suite used nominal operation and six static faults at severity 0.55 with 500 episodes per method-condition. The severity suite used six faults at severities 0.15, 0.35, and 0.75 with 200 episodes per condition. The transition suite used 18 severity-0.55 scenarios with a requested change at step 50: six onsets, six recoveries, and the directed, non-exhaustive cycle

$$\begin{array}{cc} \mathrm{dropout} & \to \mathrm{noise}\to \mathrm{ghost}\to \mathrm{clipping}\\ \; & \to \mathrm{LiDAR}\;\mathrm{latency}\to \mathrm{control}\;\mathrm{latency}\to \mathrm{dropout}. \end{array}$$

The five central methods contributed 160,500 episodes across these suites. Longer-trained and history-augmented controls, mixed and unknown degradation, five router-component ablations, and transition steps 20 and 100 contributed 262,500 comparative records.

The evaluation included four complementary experiment families addressing structural properties of the system. The interaction-matched robust baseline was evaluated under core, severity, transition, mixed/unknown, and correlated-fault protocols, contributing 42,900 episode records. The fault-policy matrix evaluated normal, robust, and six specialist policies under nominal operation and all six faults; faulty conditions used severities 0.15, 0.35, 0.55, and 0.75, contributing 60,000 episode records. Three separately executed, non-overlapping blocks contributed 24,600 additional episode records. Dedicated executed-route tracing contributed 4200 records; the deterministic label-derangement control contributed 10,800 records; and the correlated-fault control block comprising robust-100k, robust-200k, temporal-robust-200k, and diagnosed switching contributed 9600 records. The correlated-control block excluded the interaction-matched robust policy. Its 2400 correlated-fault records were counted only within the 42,900-record interaction-matched family; the shared evaluation-seed base enabled paired comparisons but did not duplicate method records. Thus, the complete evaluation record comprised 160,500+262,500+42,900+60,000+24,600=550,500 held-out simulation episode records. Identical environment and fault seeds were used within every paired method comparison. A complete experiment-family accounting is provided in Supplementary Table S6.

The correlated suite comprised burst scan dropout, moving-sector loss, autoregressive range drift, and jittered scan freeze at severities 0.35 and 0.55. These processes introduced temporal dependence and intermittency absent from atomic training faults. The mixed/unknown suite contained three simultaneous fault pairs and four untrained degradations: LiDAR bias, sector dropout, scan freeze, and action scaling. Oracle routing was undefined for mixed, unknown, and correlated conditions because no unique ground-truth specialist existed. Exact transformations, severity mappings, temporal parameters, and randomization rules for all mixed, unknown, and correlated degradations are provided in Supplementary Table S3.

Reported outcomes were success, collision, timeout, and success-weighted Euclidean path efficiency (SWEPE),

(3) $$\mathrm{SWEPE}=S\frac{L_{E}}{\max(L_{E},L)},$$

where *S* is the binary success indicator, $L_{E}$ is the start-to-goal Euclidean distance after subtracting the 0.5 m goal tolerance, and *L* is the traveled path length. SWEPE deliberately uses the same Euclidean start-to-goal reference for every method and does not require a planner-derived geodesic path, which is useful here because the controller itself is map-free and the primary comparison concerns relative closed-loop efficiency under identical procedural scenes. It preserves the success weighting and path-efficiency interpretation of SPL while avoiding an additional map/planner dependency. Accordingly, SWEPE is a study-specific metric, is not a replacement for the canonical geodesic Success weighted by Path Length (SPL) metric, and should not be compared numerically with geodesic SPL values [29]. Transition outcomes additionally included diagnosis delay, policy-route delay, acquisition coverage, switch count, and intervention count. Episodes terminating before a requested transition remained in all outcome rates. Delay was measured as the number of control steps after the observed fault transition until the first qualifying event. A qualifying event on the first post-transition observation was assigned delay zero. The same convention was used for confidence-qualified diagnosis and correct route acquisition. Episodes in which an event was not acquired were excluded from the corresponding delay mean but remained represented in acquisition coverage.

### 3.7. Statistical Analysis

The policy-training seed was the independent replication unit. Tables report the mean and sample standard deviation across seeds 42, 43, and 44. Episode records were paired by policy seed, condition or scenario, episode index, environment seed, and fault seed. Hierarchical intervals for the central dynamic comparison used B=5000 bootstrap replicates and random seed 90,000. For each replicate, policy seeds were sampled with replacement and paired episode differences were then sampled with replacement within each selected seed. Reported intervals were the 2.5th and 97.5th percentiles of the bootstrap distribution. Because only three independent training replications were available, these intervals were interpreted as episode-conditional descriptive summaries rather than population-level coverage guarantees. Seed-specific effects and leave-one-seed-out means were retained to expose sensitivity to individual training runs. Mechanism and interaction-matched comparisons report both seed-level outcomes and paired differences; episode counts were not treated as substitutes for independent training replication. Accordingly, the reported 95% intervals characterize uncertainty conditional on these three trained policy instances and the paired episode-generating process; they should not be interpreted as population-level confidence intervals over independently retrained policies.

### 3.8. Software and Reproducibility

Experiments used Python 3.8.5, NumPy 1.24.4, Gymnasium 1.0.0, PyTorch 2.4.1, Stable-Baselines3 2.4.1, SciPy 1.10.1, and Matplotlib 3.7.5 on Windows workstations. The Supplementary Materials provide the procedural environment and fault specifications, reward and training hyperparameters, degradation definitions, diagnosis-feature thresholds, routing-control details, evaluation episode accounting, and specialist cross-performance summaries reported as Tables S1–S7. Seed-level summaries, analysis outputs, frozen protocols, package-version records, model hashes, CSV-derived summaries, complete episode-level NPZ arrays, source code, and trained checkpoints were retained by the authors and are available from the corresponding author upon reasonable request. Automated validators confirmed terminal-outcome exclusivity, finite metrics, unique condition keys, expected record counts, exact compute targets within one rollout, and completion of all three policy seeds. Computational time was measured explicitly for each evaluated controller configuration. Inference was profiled in isolated single-thread CPU processes using 500 warm-up and 5000 timed valid-shape synthetic inputs per method; environment stepping was excluded. The resulting mean and 95th-percentile inference latencies are reported in Section 4.13, including the diagnosed-switching path that combines diagnosis and selected-policy inference.

## 4. Results

Results are reported in the same progression as the evaluation design. The section first quantifies held-out diagnosis and stationary-fault navigation, then examines severity dependence, longer-trained and interaction-matched monolithic baselines, and within-episode transitions. The remaining subsections examine transition sensitivity, mixed and unknown degradation, specialist specificity and routing controls, correlated processes, oracle/safety/router ablations, and computational footprint. This ordering separates outcome-level evidence from mechanism and deployment-cost analyses.

### 4.1. Held-Out Diagnosis Performance

The calibrated temporal classifier was consistent across policy seeds (Table 2). Mean accuracy was 0.836±0.011, and macro-F1 was 0.821±0.005, indicating that aggregate performance was not produced only by the nominal or other frequent class. The low ECE of 0.017±0.002 indicates well-calibrated held-out probabilities, although the calibration ablation showed that temperature scaling was not necessary for aggregate navigation success in this benchmark. Severity estimation MAE was 0.151±0.011. These figures were computed only on episode-disjoint test data.

Class-wise held-out diagnosis performance is summarized in Table 3.

**Figure 2:**
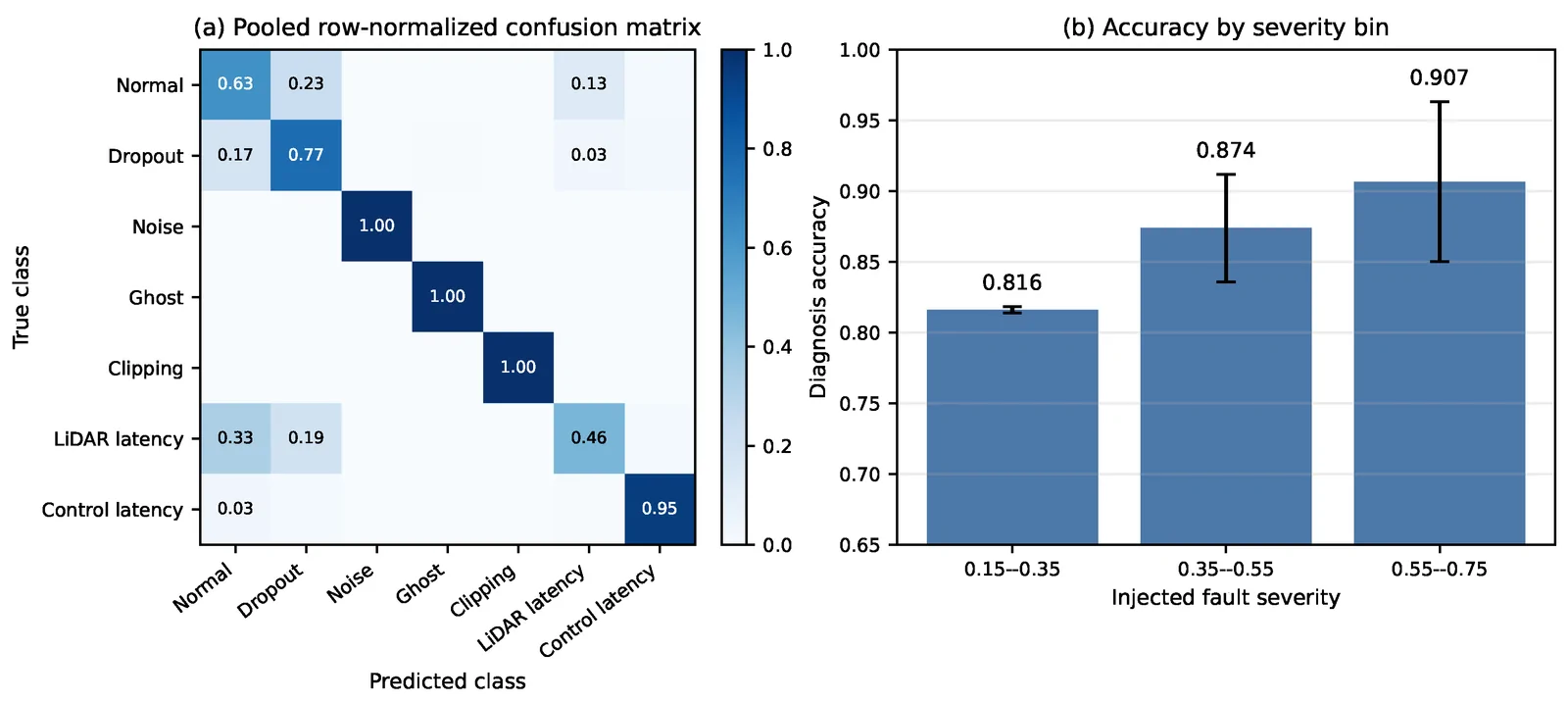
Detailed held-out diagnosis results derived from the archived test predictions. Panel (**a**) pools confusion counts across the three seed-specific test sets and normalizes each true-class row. Panel (**b**) reports seed-mean diagnosis accuracy in the fault-severity intervals [0.15,0.35), [0.35,0.55), and [0.55,0.75]; error bars show sample SD across diagnosers.

The corresponding pooled confusion structure and severity-dependent diagnosis accuracy are shown in Figure 2.

Performance was heterogeneous across classes. Clipping, noise, and ghost returns had F1 scores of at least 0.998, while dropout reached 0.666±0.031, normal operation 0.556±0.029, and LiDAR latency 0.576±0.041. LiDAR-latency recall was only 0.461±0.027. Diagnosis accuracy increased from 0.816±0.002 in the 0.15–0.35 severity bin to 0.907±0.056 in the 0.55–0.75 bin. Thus, the aggregate macro-F1 conceals a specific weakness for temporally stale but physically plausible scans and for separating mild faults from normal operation.

### 4.2. Static Fault Performance

**Figure 3:**
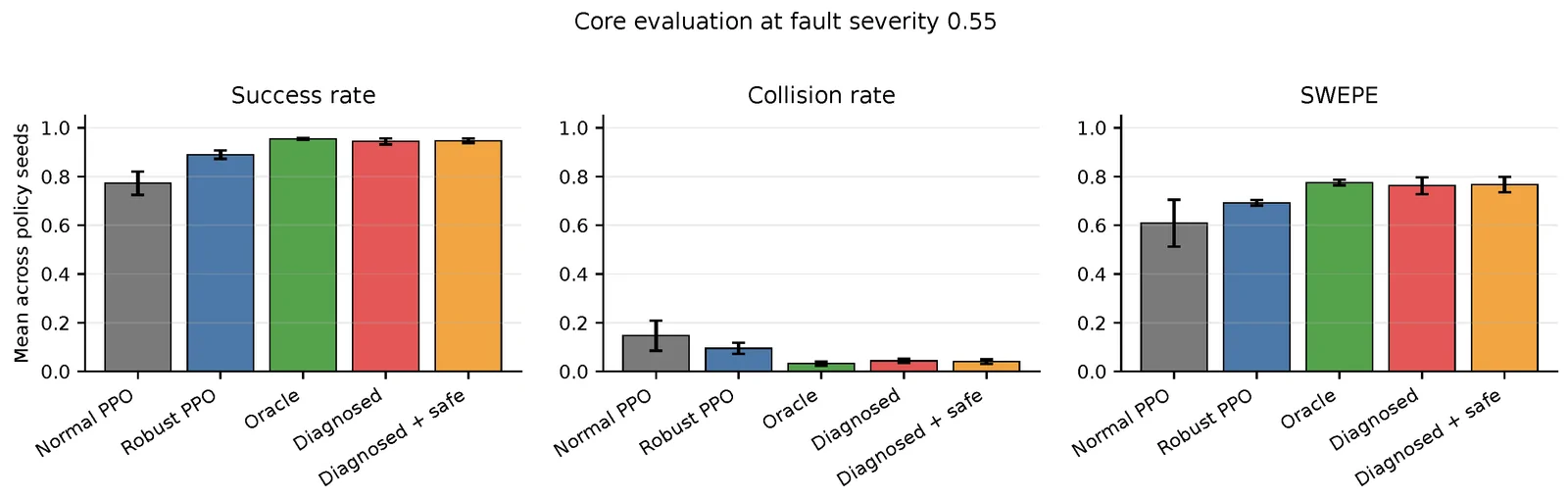
Core static-fault performance at severity 0.55 across three policy seeds. Error bars show the standard deviation of seed-level means.

Table 4 and Figure 3 summarized the severity-0.55 core evaluation. Robust PPO substantially improved over normal PPO, confirming that the monolithic baseline learned meaningful invariance. Diagnosed switching then raised success from 0.890±0.017 to 0.945±0.012 and reduced collision from 0.095±0.023 to 0.044±0.008. The mean seed-level paired differences were +5.45 percentage points in success and −5.12 percentage points in collision relative to robust PPO. Diagnosed routing remained close to the oracle routing reference (0.955±0.003 success). The safety ablation yielded only a small aggregate change, which indicated that the principal improvement came from diagnosis-aware routing rather than from stopping behavior.

### 4.3. Severity Dependence

**Figure 4:**
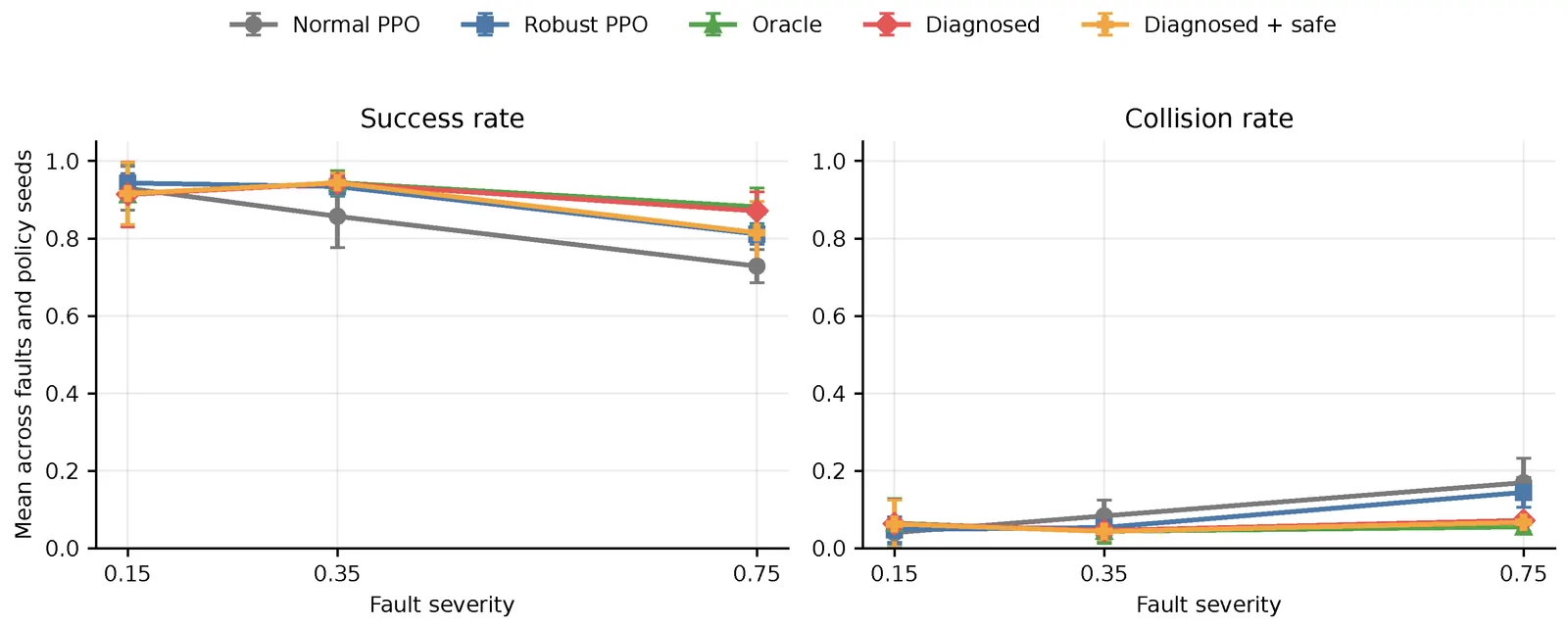
Success and collision rates across degradation severity. Lines connect seed-mean aggregate performance at each severity, and error bars show the standard deviation across policy seeds.

The severity sweep in Table 5 and Figure 4 showed a regime-dependent result. At severity 0.15, robust PPO had the highest mean success (0.943±0.025), while diagnosed switching achieved 0.914±0.084. At severity 0.35, diagnosed switching and the oracle were both approximately 0.94 and exceeded normal PPO. At severity 0.75, diagnosed switching reached 0.871±0.049, compared with 0.812±0.027 for robust PPO and 0.729±0.043 for normal PPO. Thus, routing was most useful when degradation is strong enough to require mode-specific behavior. Diagnosed-safe fell to 0.815±0.081 at severity 0.75 because conservative high-severity fallbacks can convert collision risk into non-completion. This is why the diagnosed method without the safety supervisor was retained as the central method.

### 4.4. Longer-Trained and History-Augmented Monolithic Baselines

**Figure 5:**
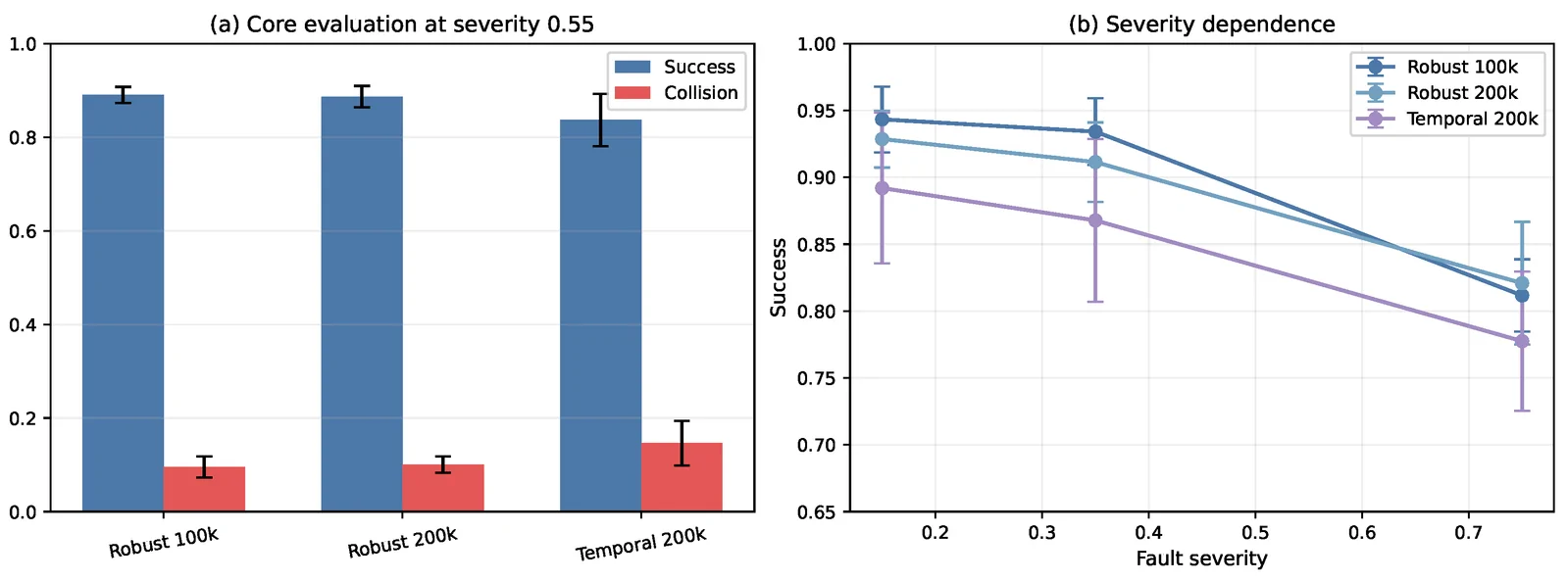
Longer-trained and history-augmented monolithic baselines. Panel (**a**) reports core success and collision; panel (**b**) reports success over severity. Error bars show sample SD across three policy-training seeds.

Table 6 and Figure 5 test whether robust-100k was disadvantaged by the tested episode budget or lack of direct history. Robust-200k did not consistently improve on robust-100k: core success changed from 0.890±0.017 to 0.887±0.023, and only severity 0.75 showed a small mean increase, from 0.812±0.027 to 0.821±0.046. The eight-frame temporal-robust-200k policy was weaker and more seed-sensitive, with 0.837±0.056 core success. Within these tested architectures and budgets, the longer training continuation and flattened temporal context did not remove the diagnosed controller’s gains. This result is specific to the tested architecture and optimization budget; it does not establish that temporal robust policies are generally inferior.

### 4.5. Interaction-Matched Robust Baseline

**Figure 6:**
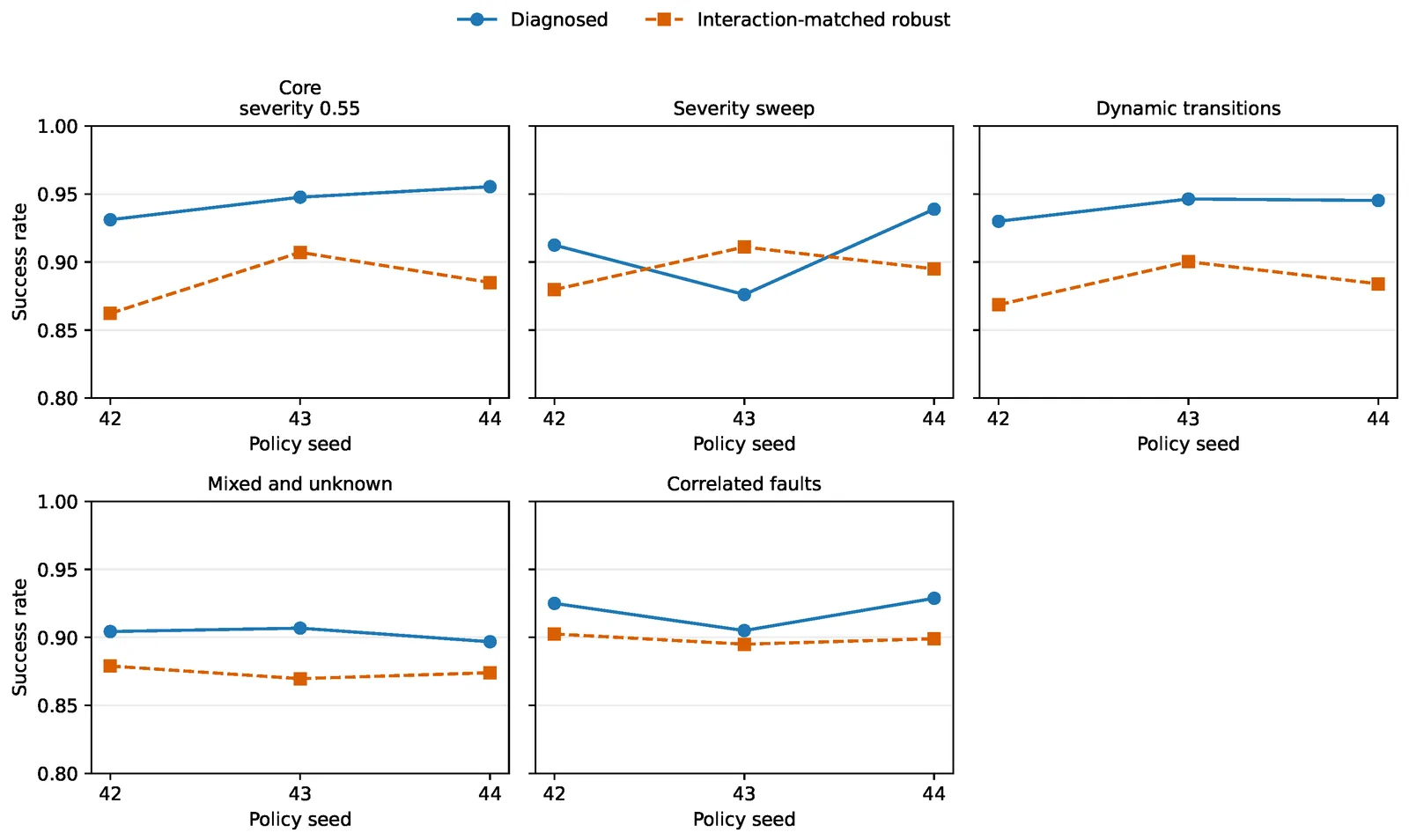
Seed-specific success for diagnosed switching and the interaction-matched monolithic robust baseline. The vertical axis is restricted to 0.80–1.00 for readability. Diagnosed switching retained higher success in every dynamic seed, while the severity sweep included a crossover inseed 43.

All interaction targets were reached within one rollout (Table 7). Table 8 and Table 9 and Figure 6 report the seed-level and aggregate outcomes of the interaction-matched baseline. Its success rates were 0.885±0.022, 0.895±0.016, 0.884±0.016, 0.874±0.005, and 0.899±0.004 in the core, severity, dynamic, mixed/unknown, and correlated suites, respectively. The mean seed-level diagnosed-minus-matched success differences were +6.00, +1.39, +5.63, +2.84, and +2.07 percentage points in those suites, respectively. In dynamic transitions, the seed-specific diagnosed-minus-matched differences were +6.14, +4.61, and +6.13 points, and collision was lower for diagnosed switching in every seed. The severity sweep contained a crossover: the matched baseline exceeded diagnosed switching by 3.50 points in seed 43, whereas diagnosed switching led in seeds 42 and 44. In mixed/unknown degradation, diagnosed success was higher, but SWEPE was 0.007 lower on average. Thus, interaction matching did not remove the aggregate success advantage under the tested protocol, but the effect was moderate and metric-dependent rather than evidence of universal architectural superiority.

### 4.6. Dynamic Fault Transitions

The central dynamic comparison contained exactly 54,000 episodes: five methods, 18 transition scenarios, 200 episodes per scenario, and three policy seeds. The seven-method comparison included robust-200k and temporal-robust-200k, producing 75,600 records across seven methods. No condition or episode was missing or duplicated. The requested step-50 transition was observed in 99.37% of diagnosed episodes, 99.43% of diagnosed-safe episodes, 99.44% of oracle episodes, 98.50% of robust-PPO episodes, and 98.15% of normal-PPO episodes. Episodes terminating before the transition remain included in all outcome rates.

**Figure 7:**
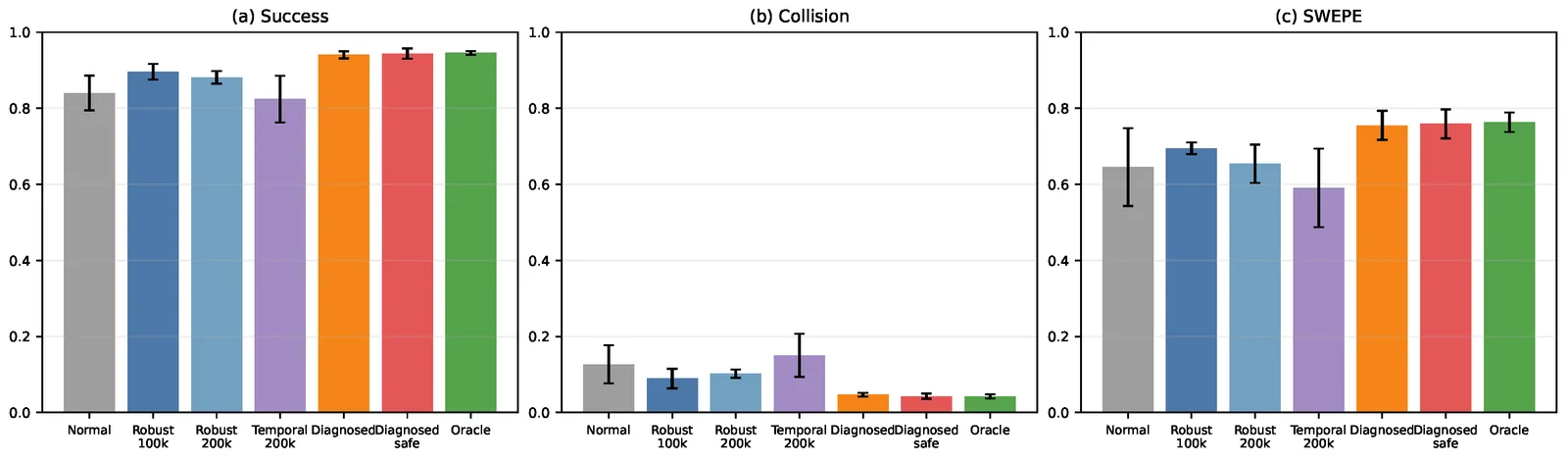
Seven-method dynamic-transition performance for all seven evaluated methods. Bars are means of seed-level outcome rates and error bars show sample SD across the three policy-training seeds.

Overall dynamic performance is shown in Table 10 and Figure 7. Under the tested budgets and architectures, diagnosed switching improved success over robust-100k by 4.44 percentage points, reduced collisions by 4.27 points, and increased SWEPE by 0.060. The corresponding episode-conditional hierarchical intervals were 2.66–7.07 success points, 1.93–7.48 fewer collision points, and 0.033–0.086 SWEPE. Relative to robust-200k, mean changes were +5.93 success points, −5.53 collision points, and +0.101 SWEPE; relative to temporal-robust-200k they were +11.64, −10.35, and +0.165, respectively. These comparisons showed that the tested longer-trained and flattened-history controls did not remove the observed advantage. The interaction-matched comparison was reported separately because it equalized the audited environment-interaction budget, while recurrent and exhaustively tuned monolithic policies remained outside the evaluated set.

The diagnosed-minus-robust dynamic effect had the same direction in every policy seed: diagnosed-minus-robust success was +0.031, +0.072, and +0.031; collision was −0.020, −0.077, and −0.031; and SWEPE was +0.032, +0.061, and +0.087 for seeds 42, 43, and 44, respectively. Omitting any one seed left a positive two-seed mean for success and SWEPE and a negative mean for collision (Table 11). This is a sensitivity check, not a substitute for additional independent training replications.

### 4.7. Transition-Type Effects and Adaptation Delay

Table 12 separates onset, recovery, and cross-fault transitions. All three transition types show positive success and SWEPE intervals and negative collision intervals relative to robust PPO. The largest success benefit occurred for cross-fault transitions, where a monolithic policy must accommodate two distinct degradation regimes within one episode. Recovery showed the smallest but still positive seed-level effect. Across the 18 individual scenarios, the unadjusted descriptive episode-conditional success interval excluded zero in the favorable direction in 10 scenarios and in the unfavorable direction in none; the analogous descriptive intervals favored collision reduction in 10 and SWEPE improvement in 12. Two non-positive success point estimates, nominal-to-LiDAR-latency (−1.0 points) and clipping-to-LiDAR-latency (−0.5 points), both had intervals spanning zero.

**Figure 8:**
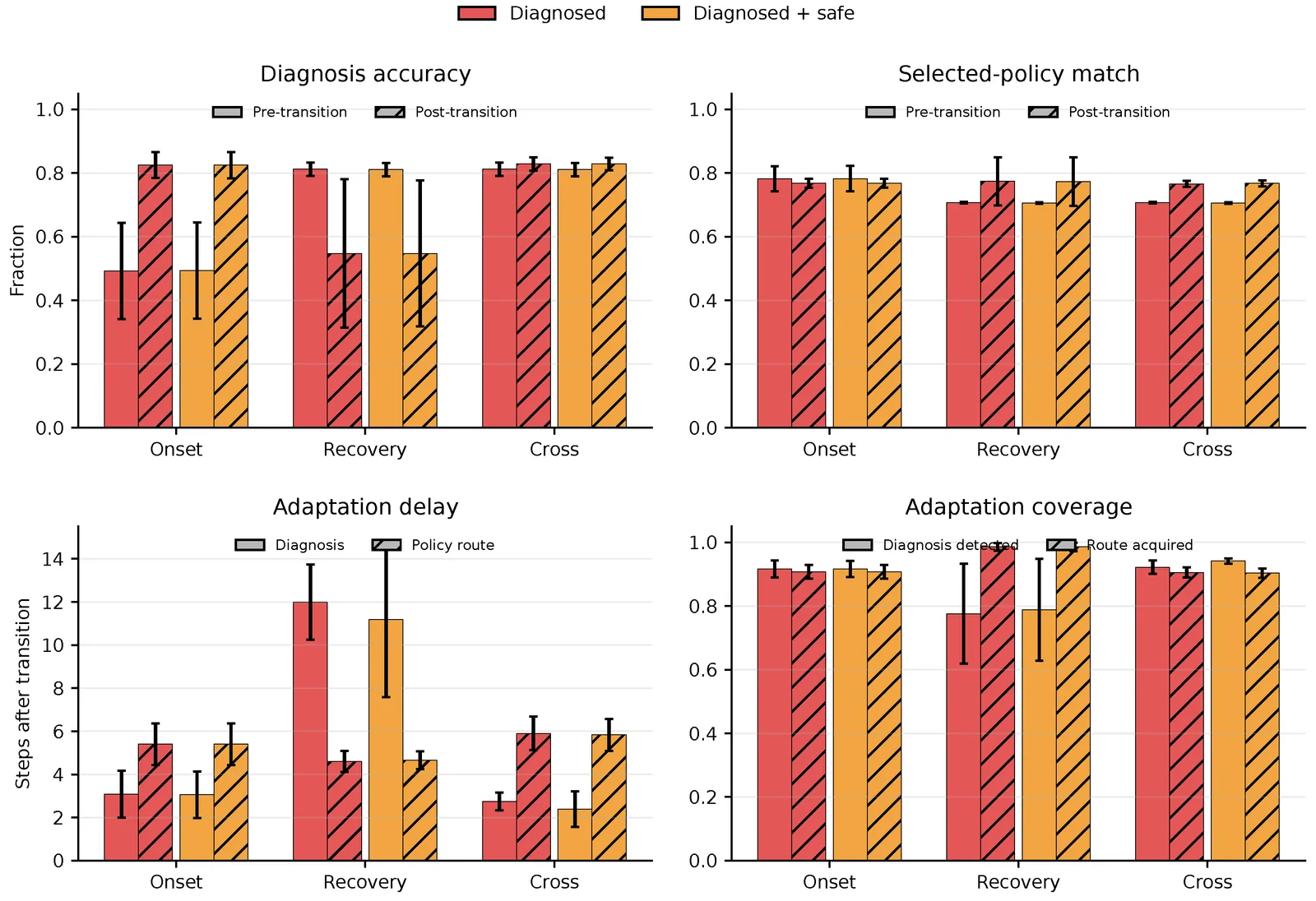
Diagnosis and policy-route adaptation after within-episode transitions. The panels distinguish delay from acquisition coverage, preventing an early route change from being misinterpreted as a confidence-qualified diagnosis.

Across all transitions, diagnosed switching required 5.55±0.71 steps to obtain a confidence-qualified correct diagnosis and 5.28±0.59 steps to acquire the correct policy route (Table 13). A qualified diagnosis was observed in 87.15%±3.72% of episodes, while the correct post-transition route was acquired in 93.29%±0.85%. Figure 8 showed that recovery to the normal class had the longest strict diagnosis delay (11.99±1.75 steps), yet the router acquired the normal route in 4.60±0.49 steps with 98.6% coverage. The difference occurred because route acquisition and the stricter confidence-qualified diagnosis criterion measured different events.

Fault-specific adaptation after nominal-to-fault onset is reported in Table 14.

Fault-specific onset results explained the aggregate delay. Noise, ghost returns, and clipping were identified rapidly, whereas dropout required 6.47±3.42 diagnosis steps and LiDAR latency required 13.25±0.60. LiDAR latency also had the lowest post-transition diagnosis accuracy (0.357±0.056) and route-match fraction (0.274±0.050). The weak LiDAR-latency scenarios therefore reflected both classifier ambiguity and delayed route acquisition rather than only dwell logic.

### 4.8. Transition-Time Sensitivity

**Figure 9:**
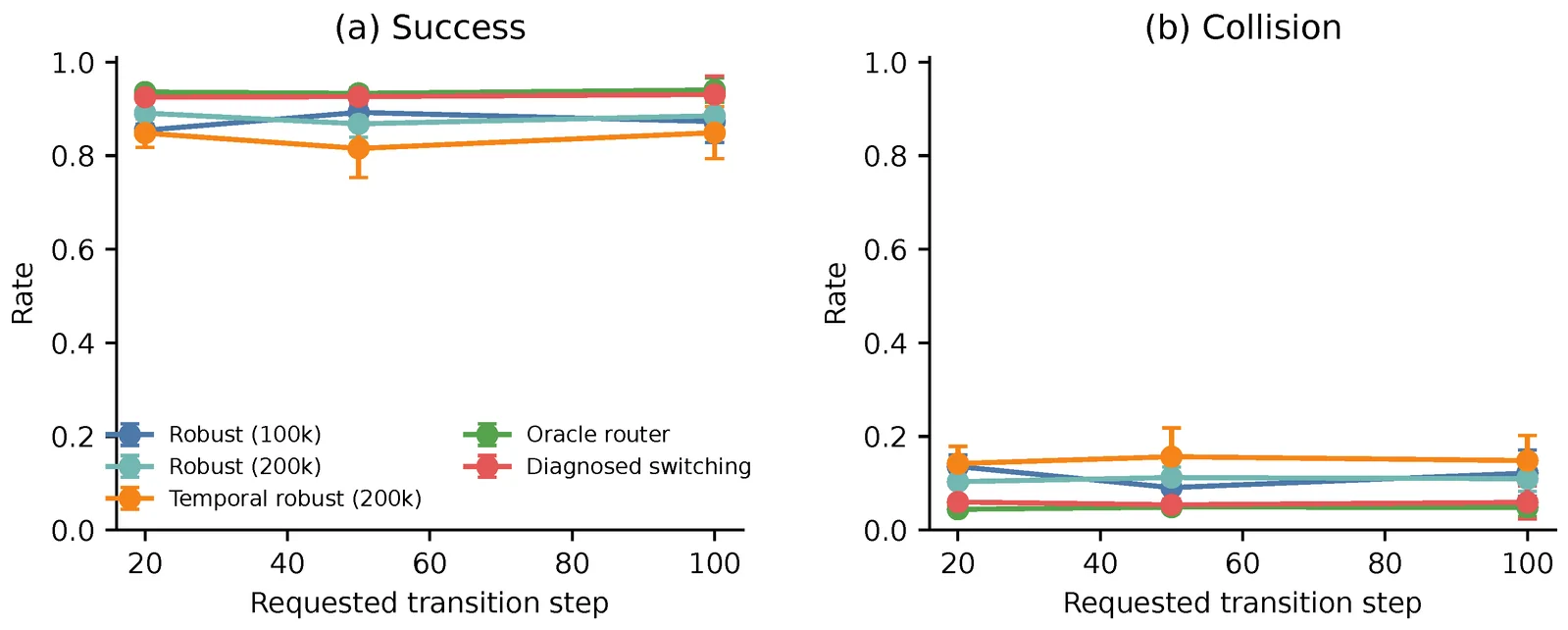
Sensitivity of success and collision to the requested within-episode transition step. Points are seed means and error bars are sample SD across three policy-training seeds. Steps 20 and 100 used dedicated evaluation-seed blocks, whereas step 50 used the first 100 episodes per scenario from the central dynamic benchmark.

Moving the requested transition from step 20 to 50 or 100 did not reverse the ranking (Table 15 and Figure 9). Diagnosed success remained between 0.925 and 0.931, while oracle remained between 0.933 and 0.940. All three monolithic baselines remained below diagnosed switching at every tested transition step. The step-20 and step-100 conditions used dedicated evaluation-seed blocks, whereas the step-50 entry reused the first 100 episodes per scenario from the 200-episode-per-scenario central dynamic benchmark. Consequently, the step-50 sensitivity estimate (0.926±0.019) represents a subset of, rather than a replication of, the central dynamic estimate (0.941±0.009). Because the three transition steps were not evaluated on a common episode-paired set, these values support robustness of the qualitative ordering rather than an episode-paired estimate of the causal effect of transition time.

### 4.9. Mixed and Unknown Degradation with Executed-Route Traces

The closed-set diagnoser was evaluated on three simultaneous fault pairs and four degradations absent from classifier training. Across both severities, diagnosed switching achieved 0.903±0.005 success and 0.087±0.009 collision, compared with 0.863±0.007 and 0.121±0.021 for robust-100k (Table 16). The mean seed-level paired diagnosed-minus-robust differences were +3.92 success points, −3.38 collision points, and +0.024 SWEPE. The advantage was concentrated in mixed faults; diagnosed and robust-100k were closer under unknown degradations. These observations indicated useful reuse of learned routes under distribution shift, not open-set identification.

**Figure 10:**
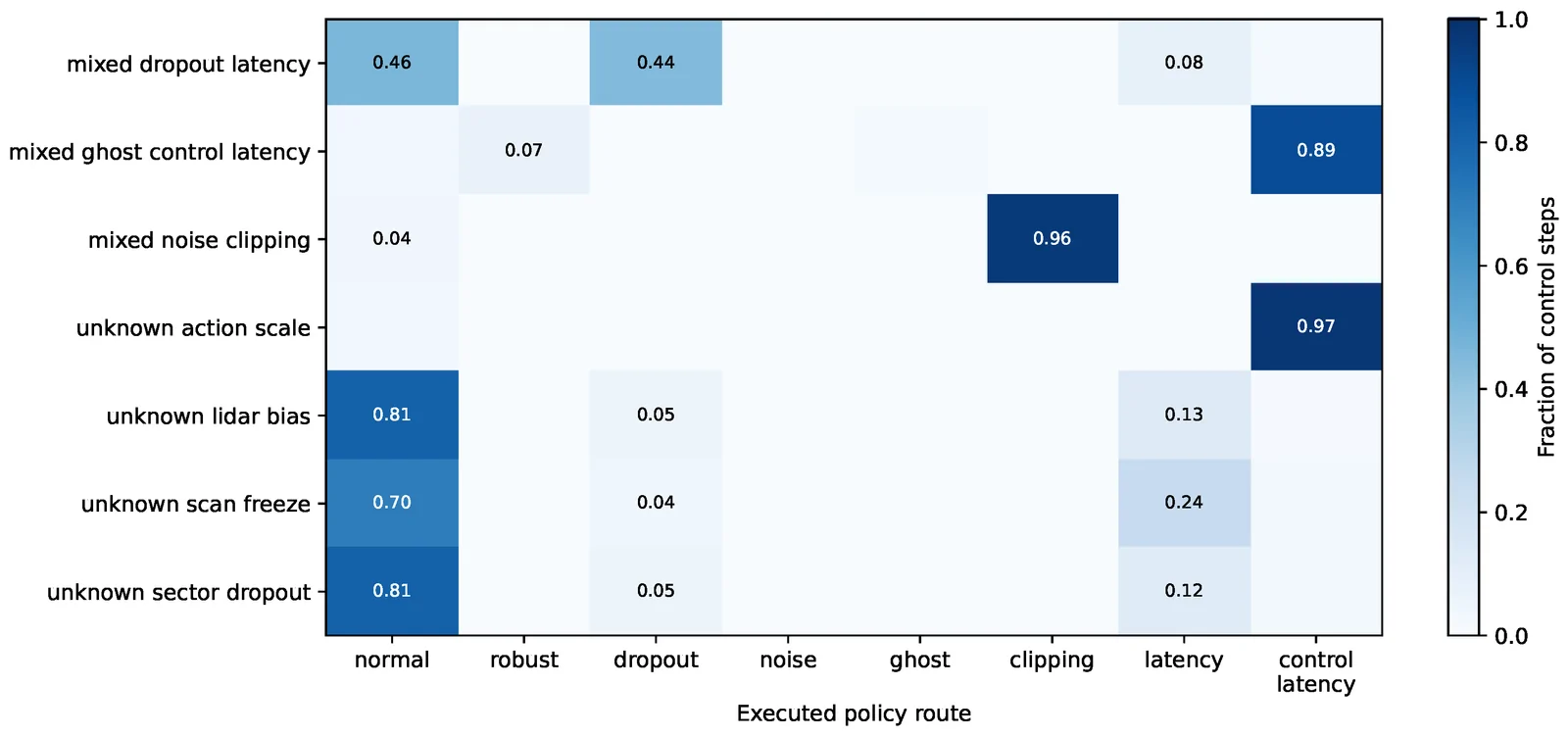
Executed policy-route fractions under mixed and unknown degradations. Each cell is the seed-mean fraction of control steps assigned to a route. Rows sum to one apart from rounding.

Executed-route traces made this reuse explicit (Figure 10). The mixed noise-clipping condition used the clipping specialist for 95.9% of control steps. Mixed ghost-control-latency used the control-latency specialist for 88.8% and the ghost specialist for 1.5%. Mixed dropout-latency split 44.2% of steps to dropout and 8.1% to LiDAR latency, while retaining the normal route for 46.0%. Among unknown degradations, action scaling was mapped predominantly to control latency (97.0%), whereas LiDAR bias, scan freeze, and sector dropout retained the normal route for 70.3–81.0% of steps. Route traces therefore showed interpretable analogical reuse rather than a hidden generic route.

### 4.10. Specialist Specificity and Routing Mechanism

**Figure 11:**
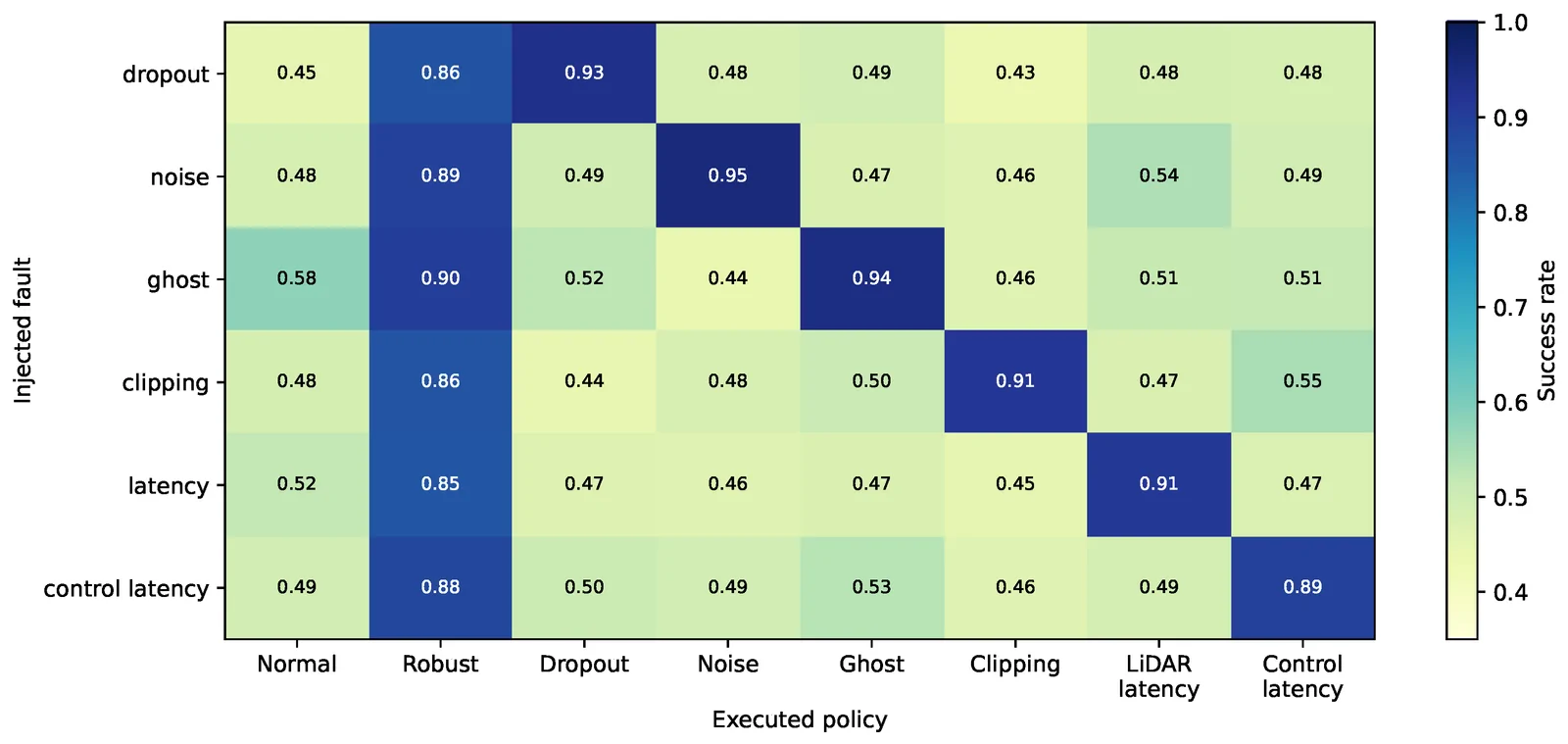
Fault-policy cross-performance at severity 0.55. Values are success rates averaged across three independently trained policy seeds. The diagonal specialist structure demonstrates mode-specific competence.

The complete cross-performance matrix characterized specialist competence independently of router behavior (Table 17 and Figure 11). Across the full four-severity cross-performance evaluation (Supplementary Table S7), fault-matched specialists achieved 0.934±0.007 success, compared with 0.510±0.016 for non-corresponding specialists and 0.872±0.012 for robust-100k. At severity 0.55, the matched specialist was the strongest in every fault row. Specialist identity therefore had a large behavioral consequence, demonstrating distinct fault-dependent competencies among the learned policies.

The deterministic label-derangement negative control retained the diagnoser architecture, confidence and severity estimators, and router hyperparameters, but permuted the non-nominal fault-to-specialist correspondence in separately executed matched-seed rollouts. Because the two controllers generated separate closed-loop trajectories, identical diagnosis and switching sequences were not assumed. On this dedicated 5400-episode-per-method mechanism-test block, standard diagnosed routing achieved 0.954±0.007 success, whereas label-deranged routing achieved 0.906±0.009 (Table 18). The paired seed-level difference was +4.80±1.42 success points, −3.70±0.86 collision points, and +0.052±0.008 SWEPE. This negative control supported a contribution of behaviorally appropriate fault-to-specialist correspondence to closed-loop performance.

### 4.11. Correlated and Intermittent Degradation

Four temporally correlated processes tested burst, moving-sector, autoregressive, and jittered-freeze behavior not present in atomic training (Table 19). Diagnosed switching achieved 0.920±0.013 success, 0.078±0.013 collision, and 0.741±0.040 SWEPE. Robust-100k achieved 0.917±0.034, 0.074±0.043, and 0.714±0.031, respectively, while the interaction-matched robust baseline achieved 0.899±0.004, 0.100±0.005, and 0.685±0.012. Diagnosed switching therefore exceeded the interaction-matched baseline by 2.07 success points and 0.056 SWEPE, with 2.17 fewer collision points, but remained close to robust-100k in success and collision. Robust-200k achieved 0.885±0.025 success, 0.113±0.023 collision, and 0.651±0.061 SWEPE, while temporal robust-200k achieved 0.900±0.072, 0.097±0.074, and 0.695±0.048, respectively. Diagnosed switching retained higher mean success and SWEPE than both 200k controls in this suite.

### 4.12. Oracle Gap, Safety, and Router Components

Compared with oracle routing, diagnosed switching differed by −0.52 success points, +0.44 collision points, and −0.008 SWEPE in the central dynamic benchmark; episode-conditional intervals included zero. This supports only a bounded descriptive statement that diagnosed routing approached the oracle outcome rate under the evaluated transition benchmark; it does not establish statistical equivalence, interchangeability, or an oracle-level guarantee outside these three trained policy instances. Diagnosed-safe changed success by +0.34 points, collision by −0.39 points, and SWEPE by +0.004 relative to diagnosed switching.

**Figure 12:**
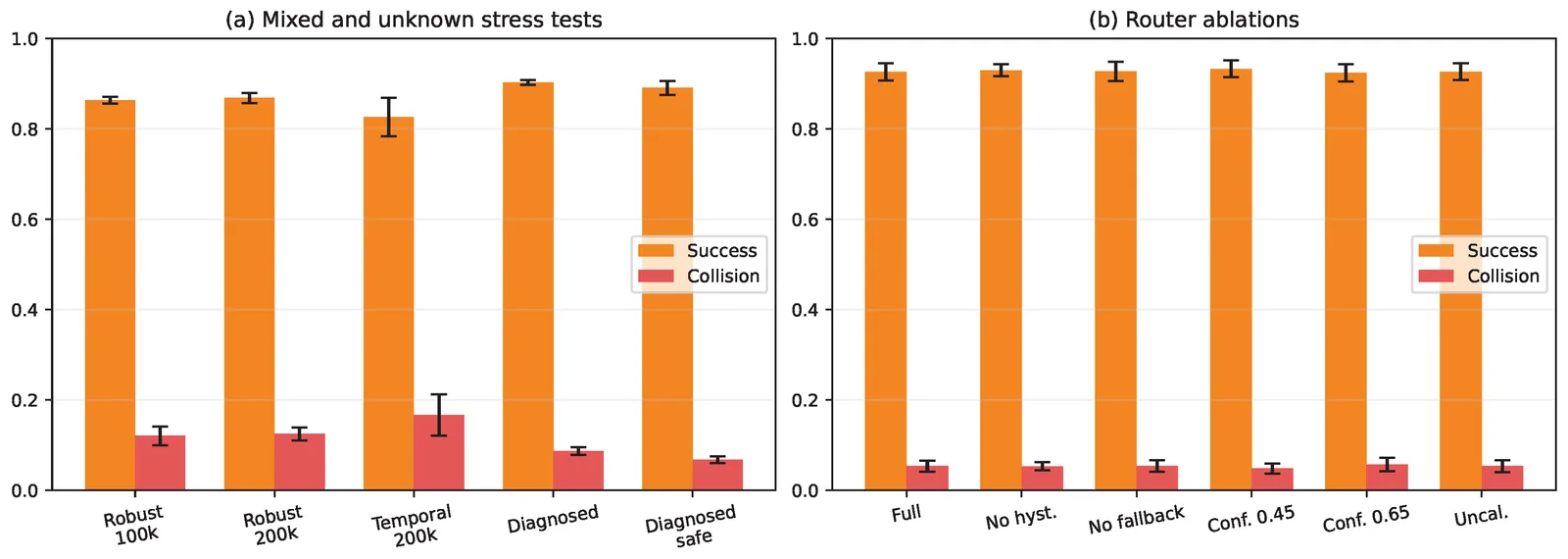
Mixed and unknown degradation performance and router-component ablations. Error bars show sample SD across policy-training seeds.

On the same first-100-episode-per-scenario step-50 subset reported in Table 15, removing hysteresis, disabling severity fallback, or setting calibration temperature to one produced seed-level outcome differences near zero. Lowering confidence from 0.55 to 0.45 increased success by 0.65 points, whereas raising it to 0.65 produced no clear aggregate change. Diagnosis and fault-matched policy selection were therefore the central mechanism in this benchmark; confidence, dwell, fallback, and calibration temperature acted as secondary implementation choices whose ablations produced comparatively small aggregate outcome changes on the matched subset. The corresponding router-component results are summarized in Table 20 and Figure 12.

### 4.13. Inference Footprint

Single-thread CPU profiling excluded simulator stepping and measured only valid-shape neural inference (Table 21). Diagnosed switching required 350.8 μs mean and 373.8 μs at the 95th percentile, compared with 115.4 and 122.9 μs for robust-100k. The complete diagnosed controller loaded 16.20 MB of model files and increased isolated-process RSS by 117.1 MB; robust-100k loaded 2.02 MB and increased RSS by 99.9 MB. The diagnosed bank therefore required about eight times the on-disk model footprint, while the measured isolated-process RSS increment was 17.2 MB larger than robust-100k; this distinction reinforces that stored model capacity and runtime process memory are related but not interchangeable resource measures. Although diagnosed inference was approximately three times slower, its measured mean remained 0.18% of the 200 ms control interval, leaving more than two orders of magnitude of timing margin within this isolated neural-inference benchmark. This isolated benchmark did not include LiDAR processing, simulator cost, operating-system jitter, or embedded hardware.

## 5. Discussion

Within the evaluated simulation benchmarks, explicit health-state estimation and specialist routing produced higher aggregate outcome rates than monolithic robust PPO in the central severity-0.55 static benchmark and the within-episode transition suites. Robust-100k already provided a strong comparator, so the observed difference was not attributable to comparison with an unprepared nominal policy. The benefit was regime-dependent rather than uniform: monolithic robustness was competitive at mild severity, whereas specialization became more useful as degradation strengthened or changed within an episode. These findings are therefore interpreted as benchmark-specific evidence for the realized diagnosis-and-routing design, not as a general superiority claim for modular control.

Interaction matching clarified the resource argument. Continuing one robust policy to the audited total environment-step budget of the policy bank produced competitive outcomes across all three seeds. Diagnosed switching retained aggregate success advantages ranging from 1.39 to 6.00 percentage points across the five evaluation suites, including 5.63 points in dynamic transitions, but one severity-sweep seed favored the matched policy and mixed/unknown SWEPE favored it slightly. Consequently, the evidence supported the realized diagnosis-and-routing system over the tested continuation protocol, not a universal claim that modularity dominates every interaction-matched monolithic learner. The policy bank also retained greater stored capacity, while active per-step capacity remained close to one PPO plus a small diagnoser.

The mechanism experiments narrowed the interpretation. Matched specialists were substantially stronger than mismatched specialists, the deterministic label-derangement negative control reduced success in every policy seed, and executed-route traces showed condition-dependent policy reuse. Together, these findings supported the importance of fault-specific specialist competence and behaviorally appropriate policy selection. The component ablations further indicated that calibration, hysteresis, fallback, and the exact confidence threshold were secondary implementation choices in this simulator.

The apparent gap between class-wise diagnosis scores and near-oracle navigation outcomes reflects the fact that these metrics operate at different levels of the closed loop. Class-wise F1 penalizes every window-level label error equally, whereas navigation success is an episode-level outcome. The router does not convert every classifier output into an immediate policy change: low-confidence predictions request the normal route, confidence-qualified class requests require three consecutive steps before switching, and the five-step dwell rule suppresses rapid reversals; only an estimated severity of at least 0.90 invokes the robust fallback immediately. Consequently, isolated or low-confidence diagnosis errors can reduce per-class F1 without necessarily changing the executed policy, and short periods on the normal or previously active route need not determine the terminal episode outcome. At the same time, the specialist cross-performance and label-derangement controls show that persistent or systematic misrouting is harmful. The small aggregate oracle gap should therefore be interpreted as closed-loop tolerance to a subset of diagnosis errors, not as evidence that diagnosis quality is unimportant or that diagnosed routing is statistically equivalent to oracle routing.

Transition experiments showed that operational adaptation depended on both classifier quality and routing logic. LiDAR latency remained the most difficult class because stale scans were physically plausible, and recovery had a longer strict confidence-qualified diagnosis delay than route acquisition. Reporting both delay and acquisition coverage prevented rapidly terminating episodes from being interpreted as successful adaptation. Qualitative ordering remained stable at requested transition steps 20, 50, and 100.

During LiDAR-latency episodes, the router did not automatically switch to the robust fallback. By design, robust fallback was invoked only when estimated severity reached 0.90. Otherwise, a low-confidence output requested the normal policy, whereas a confidence-qualified LiDAR-latency prediction had to persist for three consecutive steps and satisfy the five-step dwell logic before the latency specialist became active. A stale scan could therefore leave the controller on the normal or previously active route while confirmation was pending, lead to the latency specialist after qualified acquisition, or invoke the robust fallback only if the severity guard was triggered. The observed LiDAR-latency recall of 0.461, onset diagnosis and route delays of 13.25±0.60 and 17.78±1.31 steps, and post-transition route-match fraction of 0.274±0.050 establish that correct latency-specialist acquisition was delayed and incomplete. The recorded evaluation summaries did not separately decompose nonmatched latency steps among the alternative executed routes, so no latency-specific fallback occupancy percentage is reported. This behavior is consistent with LiDAR latency being the most difficult transition mode and with its comparatively weak scenario-level gains.

Distribution-shift tests produced a more qualified result. Diagnosed switching improved most clearly under simultaneous mixed faults, where one specialist often captured the dominant degradation. Unknown degradations were mapped to nearby known behaviors, especially action scaling to control latency, but no unknown-fault identity was inferred. This behavior follows directly from the closed-set seven-way softmax: every input must be represented through known class probabilities, and the router contains no explicit out-of-distribution score or reject class. Unknown processes whose temporal summary statistics resemble a trained fault can therefore acquire that specialist, whereas low-confidence unknown observations tend to request the normal route under the current confidence rule. The route traces should consequently be interpreted as analogical reuse of nearby learned behaviors, not as recognition of an unseen fault. For safety-critical deployment, an explicit rejection mechanism (for example, a calibrated OOD score or reject class) would be preferable to forcing all novel observations into the closed-set routing vocabulary. Under correlated and intermittent LiDAR processes, diagnosed and robust-100k had similar success and collision rates, with a modest mean SWEPE difference. Thus, the tested controller tolerated these processes without establishing broad open-set or correlated-fault superiority.

The measured inference cost was small relative to the simulator control interval but was obtained on one desktop CPU with synthetic valid-shape inputs. It quantified neural routing overhead under controlled conditions and did not establish real-time performance on an embedded robot. The larger file footprint reflected stored specialists rather than simultaneous evaluation of all policies. Immediate deployment-oriented reductions could include lightweight policy distillation, shared feature encoders, specialist pruning, or conditional parameter loading so that inactive specialists need not remain resident.

### Limitations

**Algorithmic and statistical limitations.** The diagnoser was closed-set and lacked an explicit reject option, so that novel observations could be forced into a known route. Mixed and correlated suites covered a finite catalogue rather than adversarial or continuously evolving faults, and training and evaluation layouts came from the same procedural scene distribution despite using unseen random seeds and newly sampled maps. Only three independent policy-training seeds were available. Seed-level and leave-one-seed-out effects exposed sensitivity, but three replications cannot support precise population-level uncertainty. The interaction-matched baseline used one continued PPO recipe with unchanged hyperparameters; alternative restarts, schedules, checkpoint rules, recurrent architectures, or broader tuning could change comparative performance. The temporal baseline used a flattened history rather than recurrence or attention, and the monolithic robust baselines were trained with episode-level stationary fault sampling rather than explicit within-episode transitions. A monolithic policy trained directly on transition processes could therefore narrow the observed dynamic-transition gap. Finally, the diagnoser was trained from robust-policy trajectories but deployed in a switched closed loop that also generated nominal- and specialist-policy trajectories, so policy-induced covariate shift was not isolated experimentally.

**Hardware and deployment limitations.** All fault-aware navigation evidence was simulation-only. Although the underlying nominal navigation platform was previously evaluated on physical hardware [14], those experiments did not include the fault injection, diagnosis, specialist routing, or safety mechanisms studied here and therefore do not validate the present fault-aware controller. Procedural maps and injected degradations cannot reproduce the full optical, temporal, mechanical, and safety characteristics of a physical LiDAR robot. The inference profile used one desktop CPU with synthetic valid-shape inputs and excluded environment, sensor-processing, operating-system, and embedded-hardware effects. Safety thresholds, confidence thresholds, collision margins, action limits, and fault severities remain simulator-specific engineering choices. Physical validation, explicit unknown-fault rejection, additional independent training seeds, transition-trained recurrent monolithic controls under matched interaction budgets, and policy-bank compression are therefore required before deployment-oriented or general reliability claims.

## 6. Conclusions

Fault-diagnosis-aware adaptive policy switching was evaluated for map-free mobile-robot navigation under six sensor and control degradation modes. A calibrated temporal classifier inferred health state from recent LiDAR, action, and motion histories, and a hysteretic router selected a nominal, robust-fallback, or fault-specialized PPO policy. Across the three trained policy seeds, diagnosed routing improved the central severity-0.55 static benchmark and within-episode transition outcomes over robust PPO, while the severity sweep showed regime-dependent behavior. The small aggregate gap to oracle routing is reported only as a bounded descriptive result for the evaluated transition benchmark and does not imply statistical equivalence with oracle routing.

Interaction-matched robust training did not eliminate the aggregate success advantage under the tested continuation protocol, but the comparison remains conditional on the selected training budgets, hyperparameters, and three independent training seeds. Router ablations indicated that hysteresis, fallback, calibration temperature, and nearby confidence-threshold choices had comparatively small aggregate effects on the matched subset, whereas specialist cross-performance and label derangement showed a much larger dependence on fault-matched behavior selection. The resulting architectural trade-off is therefore explicit: the method exchanges greater stored policy-bank capacity for diagnosis-conditioned specialization while retaining an active per-step footprint close to one selected PPO plus a small diagnoser and sub-millisecond profiled neural inference.

**Future work.** The next stage is physical validation of the complete fault-aware controller on a mobile robotic platform or hardware-in-the-loop testbed, including realistic sensor timing, actuation delay, compute jitter, and safety constraints. Further work should add explicit open-set/OOD rejection, increase independent training replications, compare transition-trained recurrent or attention-based monolithic policies under matched interaction budgets, and reduce policy-bank storage through distillation, shared representations, pruning, or conditional loading. These steps are necessary to determine whether the simulation-level advantages observed here persist under hardware and broader distribution shift.

Overall, the study supports diagnosis-conditioned specialist routing as a principled simulation-navigation strategy for heterogeneous degradation, while the claims remain bounded to the tested policies, training budgets, procedural environments, and simulation setting.

## Figures and Tables

**Table 1:** Archived training and capacity audit. Environment steps and PPO optimization epochs are the mean ± sample SD across seeds. The bank counts shared robust pretraining once and each specialist’s incremental fine-tuning.

Method	Env. Steps (Million)	Update Epochs	Stored Params	Active Params
Policy bank + diagnoser	136.3 ± 17.8	42,913 ± 4252	1,316,416	165,917
Robust PPO (100k)	20.8 ± 3.1	12,670 ± 1917	164,357	164,357
Robust PPO (200k)	39.2 ± 4.1	23,940 ± 2512	164,357	164,357
Temporal robust PPO (200k)	49.9 ± 15.8	30,430 ± 9626	503,045	503,045

**Table 2:** Held-out diagnosis performance with episode-disjoint splits. Values in the final row are the mean ± sample SD across the three policy seeds.

Seed	Accuracy	Macro-F1	ECE	Severity MAE
42	0.847	0.824	0.018	0.142
43	0.826	0.815	0.017	0.163
44	0.835	0.825	0.015	0.148
Mean ± SD	0.836±0.011	0.821±0.005	0.017±0.002	0.151±0.011

**Table 3:** Class-wise held-out diagnosis performance. Values are mean ± sample SD across the three independently trained diagnosers.

Class	Precision	Recall	F1
Clipping	1.000±0.000	1.000±0.000	1.000±0.000
Control latency	0.963±0.061	0.947±0.025	0.954±0.028
Dropout	0.638±0.180	0.757±0.157	0.666±0.031
Ghost	0.997±0.004	0.999±0.002	0.998±0.001
LiDAR latency	0.769±0.074	0.461±0.027	0.576±0.041
Noise	0.999±0.002	0.999±0.001	0.999±0.001
Normal	0.512±0.042	0.631±0.153	0.556±0.029

**Table 4:** Core evaluation at fault severity 0.55. Values are mean ± sample SD across three policy seeds and aggregate nominal operation plus six fault modes.

Method	Success	Collision	Timeout	SWEPE
Normal PPO	0.773±0.048	0.147±0.062	0.080±0.015	0.609±0.096
Robust PPO	0.890±0.017	0.095±0.023	0.015±0.015	0.692±0.011
Oracle	0.955±0.003	0.032±0.008	0.012±0.006	0.776±0.011
Diagnosed	0.945±0.012	0.044±0.008	0.011±0.006	0.762±0.034
Diagnosed-safe	0.948±0.010	0.040±0.009	0.012±0.006	0.767±0.032

**Table 5:** Success rate in the static severity sweep. Values are the mean ± sample SD across three policy seeds and aggregate the six fault modes.

Method	Severity 0.15	Severity 0.35	Severity 0.75
Normal PPO	0.930±0.057	0.857±0.080	0.729±0.043
Robust PPO	0.943±0.025	0.934±0.025	0.812±0.027
Oracle	0.913±0.081	0.944±0.031	0.882±0.048
Diagnosed	0.914±0.084	0.942±0.022	0.871±0.049
Diagnosed-safe	0.916±0.081	0.944±0.025	0.815±0.081

**Table 6:** Longer-trained and history-augmented comparison of monolithic robust baselines. Values are mean ± sample SD across three policy-training seeds. Core aggregates nominal operation and six faults at severity 0.55; severity columns aggregate six faults.

Method	Core	Severity 0.15	Severity 0.35	Severity 0.75
Robust PPO (100k)	0.890±0.017	0.943±0.025	0.934±0.025	0.812±0.027
Robust PPO (200k)	0.887±0.023	0.929±0.021	0.911±0.030	0.821±0.046
Temporal robust PPO (200k)	0.837±0.056	0.892±0.056	0.868±0.061	0.777±0.052

**Table 7:** Interaction-budget matching for the continued monolithic robust PPO baseline. Each target equals the audited total environment interactions used by the corresponding policy bank and diagnoser; overshoot is less than one 16,384-step rollout.

Policy Seed	Bank Target Steps	Matched Achieved Steps	Overshoot
42	153,688,672	153,700,000	11,328
43	137,150,288	137,160,000	9712
44	118,196,080	118,200,000	3920

**Table 8:** Outcome rates for diagnosed switching and the interaction-matched monolithic robust PPO baseline. Values are mean ± sample SD across three policy-training seeds. D: diagnosed; M: interaction-matched.

Suite	Success D	Success M	Collision D	Collision M
Core (0.55)	0.945±0.012	0.885±0.022	0.044±0.008	0.105±0.015
Severity sweep	0.909±0.032	0.895±0.016	0.061±0.030	0.097±0.011
Dynamic	0.941±0.009	0.884±0.016	0.047±0.005	0.106±0.009
Mixed/unknown	0.903±0.005	0.874±0.005	0.087±0.009	0.118±0.008
Correlated	0.920±0.013	0.899±0.004	0.078±0.013	0.100±0.005

**Table 9:** Path efficiency and paired seed-level differences for diagnosed switching minus the interaction-matched robust baseline. Method entries report mean ± sample SD across three policy-training seeds. Difference entries report the mean ± sample SD of the three seed-level paired differences. Success and collision differences are expressed in percentage points; SWEPE is unitless.

Suite	SWEPE D	SWEPE M	ΔSuccess	ΔCollision	ΔSWEPE
Core (0.55)	0.762±0.034	0.680±0.026	+6.00±1.68	−6.14±0.84	+0.083±0.036
Severity sweep	0.742±0.053	0.693±0.032	+1.39±4.27	−3.63±4.06	+0.050±0.067
Dynamic	0.755±0.038	0.680±0.024	+5.63±0.88	−5.91±0.36	+0.075±0.041
Mixed/unknown	0.664±0.048	0.671±0.007	+2.84±0.77	−3.10±0.92	−0.007±0.050
Correlated	0.741±0.040	0.685±0.012	+2.07±1.00	−2.17±1.00	+0.056±0.042

**Table 10:** Seven-method step-50 dynamic-transition benchmark. Values are mean ± sample SD across three policy-training seeds and 18 scenarios.

Method	Success	Collision	Timeout	SWEPE
Normal PPO	0.840±0.046	0.127±0.050	0.033±0.013	0.646±0.102
Robust PPO (100k)	0.896±0.020	0.090±0.026	0.014±0.012	0.695±0.015
Robust PPO (200k)	0.881±0.017	0.102±0.011	0.017±0.014	0.654±0.050
Temporal robust PPO (200k)	0.824±0.061	0.150±0.057	0.025±0.026	0.591±0.103
Oracle router	0.946±0.005	0.042±0.005	0.012±0.005	0.764±0.026
Diagnosed switching	0.941±0.009	0.047±0.005	0.013±0.005	0.755±0.038
Diagnosed-safe	0.944±0.013	0.043±0.007	0.013±0.007	0.759±0.038

**Table 11:** Seed-level effects for the central dynamic comparison (diagnosed minus robust PPO). The final column is the range of two-seed means obtained by omitting one policy seed.

Metric	Seed 42	Seed 43	Seed 44	Leave-One-Seed-Out Range
Success	+0.031	+0.072	+0.031	[+0.031, +0.051]
Collision	−0.020	−0.077	−0.031	[−0.054, −0.026]
SWEPE	+0.032	+0.061	+0.087	[+0.047, +0.074]

**Table 12:** Paired dynamic performance differences for diagnosed switching minus robust PPO. Brackets show hierarchical 95% intervals conditional on the three archived policy seeds. Success and collision are in percentage points; SWEPE is unitless.

Transition	ΔSuccess (pp)	ΔCollision (pp)	ΔSWEPE
Overall	+4.44 [2.66, 7.07]	−4.27 [−7.48, −1.93]	+0.060 [0.033, 0.086]
Onset	+4.58 [2.50, 6.89]	−4.47 [−7.31, −2.25]	+0.070 [0.051, 0.090]
Recovery	+2.89 [1.11, 4.81]	−2.25 [−4.50, −0.14]	+0.045 [0.005, 0.086]
Cross-fault	+5.86 [3.06, 9.56]	−6.08 [−10.53, −2.75]	+0.065 [0.040, 0.085]

**Table 13:** Diagnosis-and-routing adaptation after a dynamic transition. Values are mean ± sample SD across policy seeds. Delays are in control steps.

Transition	Diagnosis Delay	Route Delay	Diagnosis Coverage	Route Coverage
Onset	3.08±1.09	5.40±0.97	0.917±0.026	0.908±0.021
Recovery	11.99±1.75	4.60±0.49	0.776±0.157	0.986±0.012
Cross-fault	2.75±0.40	5.90±0.78	0.922±0.021	0.905±0.016

**Table 14:** Fault-specific adaptation after nominal-to-fault onset. Values are the mean ± sample SD across policy seeds and use episodes that reached the requested transition. Delays are in control steps.

Fault	Diagnosis Delay	Route Delay	Post-Diagnosis acc.	Post-Route Match
Dropout	6.47±3.42	8.54±3.78	0.663±0.235	0.540±0.100
Noise	0.01±0.01	2.18±0.11	0.998±0.001	0.960±0.005
Ghost	0.01±0.02	2.17±0.07	1.000±0.000	0.967±0.002
Clipping	0.56±0.48	2.55±0.78	0.991±0.008	0.954±0.003
LiDAR latency	13.25±0.60	17.78±1.31	0.357±0.056	0.274±0.050
Control latency	2.13±2.59	4.70±2.58	0.945±0.025	0.907±0.015

**Table 15:** Sensitivity to the requested transition step. Each cell is the success rate, reported as the mean ± sample SD across policy-training seeds, with 100 episodes per scenario. Steps 20 and 100 used dedicated evaluation-seed blocks; step 50 used the first 100 episodes per scenario from the 200-episode-per-scenario central dynamic benchmark.

Method	Step 20	Step 50	Step 100
Robust PPO (100k)	0.854±0.013	0.892±0.018	0.873±0.045
Robust PPO (200k)	0.891±0.006	0.868±0.029	0.885±0.030
Temporal robust PPO (200k)	0.848±0.030	0.815±0.062	0.849±0.056
Oracle router	0.936±0.007	0.933±0.008	0.940±0.026
Diagnosed switching	0.925±0.011	0.926±0.019	0.931±0.040

**Table 16:** Held-out mixed/unknown-fault stress test at severities 0.35 and 0.55. Values are mean ± sample SD across three policy-training seeds.

Method	Success	Collision	Timeout	SWEPE
Robust PPO (100k)	0.863±0.007	0.121±0.021	0.016±0.015	0.640±0.056
Robust PPO (200k)	0.868±0.011	0.125±0.014	0.006±0.003	0.641±0.052
Temporal robust PPO (200k)	0.826±0.042	0.166±0.046	0.007±0.004	0.614±0.066
Diagnosed switching	0.903±0.005	0.087±0.009	0.010±0.005	0.664±0.048
Diagnosed-safe	0.891±0.015	0.068±0.008	0.041±0.019	0.660±0.044

**Table 17:** Fault-policy cross-performance at severity 0.55. Entries are success rates averaged across three policy seeds; bold values identify the fault-matched specialist in each row.

Fault	Normal	Robust	Dropout	Noise	Ghost	Clipping	LiDAR Latency	Control Latency
Dropout	0.447	0.860	**0.930**	0.477	0.490	0.433	0.483	0.480
Noise	0.480	0.893	0.493	**0.950**	0.473	0.463	0.540	0.490
Ghost	0.577	0.900	0.523	0.437	**0.940**	0.460	0.510	0.513
Clipping	0.483	0.857	0.440	0.477	0.503	**0.913**	0.473	0.547
Latency	0.517	0.853	0.467	0.460	0.473	0.447	**0.910**	0.470
Control Latency	0.490	0.880	0.500	0.490	0.530	0.460	0.487	**0.893**

**Table 18:** Deterministic label-derangement negative control on a dedicated severity-0.55, step-50 dynamic block comprising 18 scenarios and 100 episodes per scenario and policy seed. Each method therefore contained 5400 matched episodes across three policy seeds. Environment and fault seeds were paired between routing conditions, but the controllers generated separate closed-loop trajectories. Values are mean ± sample SD across policy seeds. Differences are standard diagnosed routing minus label-deranged routing.

Method or Difference	Success	Collision	SWEPE
Diagnosed routing	0.954±0.007	0.042±0.005	0.756±0.036
Label-deranged routing	0.906±0.009	0.079±0.011	0.704±0.039
Paired difference	+4.80±1.42 pp	−3.70±0.86 pp	+0.052±0.008

**Table 19:** Performance under temporally correlated and intermittent LiDAR degradations. Values are mean ± sample SD across three policy seeds and aggregate four held-out conditions at severities 0.35 and 0.55.

Method	Success	Collision	SWEPE
Robust-100k	0.917±0.034	0.074±0.043	0.714±0.031
Robust-200k	0.885±0.025	0.113±0.023	0.651±0.061
Temporal robust-200k	0.900±0.072	0.097±0.074	0.695±0.048
Interaction-matched robust	0.899±0.004	0.100±0.005	0.685±0.012
Diagnosed switching	0.920±0.013	0.078±0.013	0.741±0.040

**Table 20:** Router ablations on matched step-50 dynamic episodes (100 episodes per scenario and policy seed). The full-router condition is the same first-100-episode-per-scenario subset reported for step 50 in Table 15. Values are the mean ± sample SD across policy-training seeds.

Variant	Success	Collision	Timeout	SWEPE
Full router	0.926±0.019	0.054±0.012	0.020±0.009	0.739±0.038
No hysteresis	0.930±0.014	0.053±0.009	0.017±0.010	0.745±0.019
No severity fallback	0.927±0.021	0.054±0.013	0.019±0.009	0.740±0.039
Confidence 0.45	0.933±0.019	0.048±0.011	0.019±0.011	0.749±0.028
Confidence 0.65	0.924±0.019	0.057±0.015	0.019±0.006	0.733±0.045
Uncalibrated confidence	0.926±0.019	0.054±0.013	0.020±0.008	0.738±0.040

**Table 21:** Single-thread CPU inference profile using 500 warm-up and 5000 timed synthetic valid-shape inputs per method. Environment stepping was excluded. RSS increments were measured in isolated processes relative to the imported-process baseline.

Method	Mean (μs)	P95 (μs)	Loaded Files (MB)	RSS Increment (MB)
Robust-100k	115.4	122.9	2.02	99.9
Robust-200k	115.7	118.0	2.02	100.3
Temporal robust-200k	147.5	155.1	6.17	109.1
Diagnosed switching	350.8	373.8	16.20	117.1

## Data Availability

The Supplementary Materials include the methodological specifications and summary results reported as Tables S1–S7. The underlying episode-level data, derived analysis files, source code, trained checkpoints, seed-level summaries, and integrity records are not included in the submission package owing to their size and are available from the corresponding author upon reasonable request.

## Supplementary Materials

The following supporting information can be downloaded at https://www.mdpi.com/article/10.3390/s26206452/s1, Table S1: Procedural Environment and Scene Generation; Table S2: Reward and PPO Hyperparameters; Table S3: Mixed, Unknown, and Correlated Degradations; Table S4: Diagnosis-Feature Thresholds; Table S5: Deterministic label-derangement mapping; Table S6: Evaluation Episode Accounting; Table S7: All-Severity Specialist Cross-Performance Summary.
