# Supplementary material for: Fault-Diagnosis-Aware Adaptive Policy Switching for Robust Map-Free Mobile Robot Navigation Under Sensor and Control Degradation
Source: Sensors (Basel). 2026 Oct 12;26(20):6452. doi: 10.3390/s26206452 (PMC13708949; doi:10.3390/s26206452)
Supplement: Supplementary file 1 [file sensors-26-06452-s001.zip › sensors-4524764-supplementary.pdf]

Supplementary Methods and Results

Fault-Diagnosis-Aware Adaptive Policy Switching for Robust Map-Free Mobile Robot Navigation under Sensor and Control Degradation

Taner Yilmaz and Omur Aydogmus

This document reports supplementary implementation and analysis details for the procedural environment, reward and policy training, degradation processes, diagnostic features, deterministic label-derangement control, evaluation episode accounting, and all-severity specialist comparison. Symbols follow the main article. The sensor span was $R = r_{\max} - r_{\min} = 0.84$ m and normalized severity was $s \in [0, 1]$.

Table S1. Procedural Environment and Scene Generation

Parameter	Implemented specification
Workspace	Square, 10×10 m; pose coordinates were clipped to $[-4.70, 4.70]$ m on both axes.
Internal walls	Eight axis-aligned rectangles per episode, each 0.30 m thick: four horizontal lengths $\{3.00, 2.00, 2.00, 1.25\}$ m and four vertical lengths $\{1.00, 2.00, 1.50, 1.25\}$ m.
Wall placement	Wall coordinates were sampled uniformly inside the workspace. Candidate wall centers had to remain at least 1.0 m from existing wall centers. A wall was rejected when the start pose lay inside its rectangle expanded by 0.30 m.
Start pose	$x, y \sim \mathcal{U}[-3, 3]$ m and heading $\theta \sim \mathcal{U}[0, 2\pi)$.
Target	$x, y \sim \mathcal{U}[-4.3, 4.3]$ m; Euclidean start–target distance at least 3.0 m; target rejected when inside any wall rectangle expanded by 0.30 m. No explicit global-connectivity test was applied.
Robot/collision model	Differential-drive point pose for integration. Collision was defined by simulator-ground-truth clean LiDAR clearance below 0.17 m; no separate polygonal footprint was used.
Kinematics	Wheel diameter 66 mm; axle length 160 mm; $v = 0.11(a^v + 1)$ m/s and $\omega = 2.84a^\omega$ rad/s for normalized actions in $[-1, 1]^2$; control interval 0.2 s.
LiDAR	120 beams uniformly spanning $[0, 2\pi)$ from the robot center; valid interval $[0.16, 1.00]$ m. Each ray tested 100 points from 0.01 to 1.00 m against axis-aligned wall rectangles.
Termination	Goal distance below 0.50 m; clean clearance below 0.17 m; or 1000 control steps.
Speed guard	Enabled for all training roles. When degraded pre-step minimum range was below 0.22 m, normalized linear action was multiplied by 0.65 before kinematic conversion.
Train/test layout relation	Training and evaluation used the same procedural generator with disjoint random seeds and newly sampled layouts.

Table S2. Reward and PPO Hyperparameters

For a nonterminal, noncollision step, the fixed shaping reward was

$$r_t = k_d(2d_t)^{-1} + k_t(t/1000) + k_\omega|\omega_t| + k_{\Delta d}\Delta d_{t,5} + k_v v_t + k_c \max(0, 0.23 - c_t^{\mathrm{clr}}) + k_h \cos(e_t) + k_s\|a_t - a_{t-1}\|_2^2.$$

Here d_t was goal distance, $\Delta d_{t,5}$ was the distance difference across the five-sample buffer, c_t^{clr} was clean minimum clearance, e_t was heading error, and D_{travel} denotes the accumulated traveled path length.

Item	Value	Implementation
Goal / collision	+1.0 / −1.0	Collision replaced other step reward; goal reward was additive when collision was absent.
Timeout	−0.60	Applied at 1000 steps and constrained to $[-1.0, -0.5]$.
k_d	0.0006	Inverse-distance term $(2d_t)^{-1}$.
k_t	−0.00035	Normalized step fraction $t/1000$.
k_ω	−0.00018	Absolute angular velocity.
$k_{\Delta d}$	−0.0004	Five-sample distance difference.
k_v	0.00022	Linear velocity.
k_c	−0.0010	Danger exposure $\max(0, 0.23 - c_t^{\mathrm{clr}})$.
k_h	0.0008	Cosine of target-heading error.
k_s	−0.0020	Squared normalized action change.
Terminal efficiency	$k_e = 0.01$	At goal or timeout, $\mathrm{clip}\{k_e/\max[10^{-3}, \lvert D_{\mathrm{travel}} - L_E \rvert], -0.2, 0.2\}$.
PPO architecture	Actor 256–256–128; critic 128–128	Multilayer perceptrons.
Rollout / batch	1024 / 256	Sixteen subprocess environments.
γ / GAE λ	0.9903838 / 0.9851848	Common to all policy roles.
Nominal/robust PPO	LR 5.129×10^{-4}; clip 0.225; 10 epochs	100,000 episodes from scratch per seed.
Specialist fine-tuning	LR 10^{-4}; clip 0.12; 5 epochs; target KL 0.03	100,000 episodes initialized from the seed-matched robust checkpoint.
Robust-200k	Same optimizer and PPO settings as robust-100k	Continued from the seed-matched robust-100k checkpoint to 200,000 cumulative episodes.
Temporal-robust-200k	Same PPO settings; flattened eight-observation input	Trained from scratch for 200,000 episodes.
Interaction-matched robust	Same randomization and PPO settings as robust-100k	Continued from robust-200k until the seed-specific audited interaction target; the final excess was below one 16,384-step rollout.
Checkpoint rule	Final checkpoint	No test-performance-based checkpoint selection.

Table S3. Mixed, Unknown, and Correlated Degradations

All output ranges were clipped to $[0.16, 1.00]$ m. Integer random draws used the half-open upper bound of NumPy's `integers` implementation.

Condition	Transformation and severity mapping	Temporal/random parameters
Dropout + latency	Clean scan delayed by $d = \max(1, \mathrm{round}(1 + 7s))$, then per-beam dropout with probability $0.05 + 0.70s$ to 1.0 m.	Same episode history rule as the atomic latency fault.
Noise + clipping	Gaussian $\mathcal{N}(0, (0.20sR)^2)$ followed by cap $1.0 - 0.65sR$.	Independent beam noise each step.
Ghost + control latency	Per-beam ghost probability $0.05 + 0.55s$ with replacement $\mathcal{U}[0.16, 0.16 + 0.30R]$; requested action delayed by d.	Sensor and action processes applied together.
LiDAR bias	$r'_i = r_i + 0.20sR$.	Constant additive bias within a step.

Condition	Transformation and severity mapping	Temporal/random parameters
Sector dropout	A contiguous sector of round$[120(0.10 + 0.35s)]$ beams was set to 1.0 m.	Sector start sampled once per episode.
Scan freeze	Most recently updated scan was repeated.	Update period $\max(2, \text{round}(10 - 6s))$ steps.
Action scaling	$a^{v'} = -1 + (1 - 0.65s)(a^v + 1)$; $a^{\omega'} = (1 - 0.65s)a^{\omega}$.	Applied every step.
Burst dropout	Burst start probability $0.015 + 0.10s$; active-scan beam dropout probability $0.45 + 0.50s$.	Burst length sampled from integers $[2, \max(3, \text{round}(3 + 10s)))$.
Moving sector loss	Contiguous width round$[120(0.10 + 0.35s)]$ set to 1.0 m.	Sector increment $\mathcal{N}(0.4 + 1.8s, 0.35)$ beams/step with circular wrap.
AR(1) range drift	Global bias $b_t = 0.94b_{t-1} + \epsilon_t$ plus spatially smoothed beam noise; $\epsilon_t \sim \mathcal{N}(0, [R(0.008 + 0.045s)]^2)$.	Beam noise used the same scale and a nine-beam moving-average kernel.
Jittered freeze	A held scan was repeated after a start event of probability $0.02 + 0.13s$.	Hold duration sampled from integers $[1, \max(2, \text{round}(2 + 9s)))$.
Delay initialization	Delay histories were cleared at reset; initial delayed outputs used the earliest valid scan or earliest requested action.	Deterministic and identical in training/evaluation.

Table S4. Diagnosis-Feature Thresholds

Feature	Fixed definition
Near maximum	$r_i \geq r_{\max} - 0.01R$.
Near minimum	$r_i \leq r_{\min} + 0.01R$.
Saturation	Normalized range below 0.05 or above 0.95.
Large temporal jump	$\lvert r_{t,i} - r_{t-1,i} \rvert / R > 0.20$.
Temporal correlation	Pearson correlation of consecutive scans; set to zero when either scan standard deviation was at most 10^{-6}; first-frame value was one.
History	Eight scans/actions/motions; diagnosis-dataset sample stride two.
Severity-regression targets	Zero for nominal windows and the injected episode-level severity for faulty windows; the least-squares fit used all training windows.
Severity output	Linear least-squares regression output clipped to $[0, 1]$ before routing.
Calibration	Temperature selected by minimum negative log likelihood on validation episodes from 81 logarithmically spaced candidates in $[0.25, 4]$; ECE used ten equal-width confidence bins over $[0, 1]$.

Table S5. Deterministic label-derangement mapping

Accepted diagnosis	Routed specialist
Dropout	Noise
Noise	Ghost
Ghost	Range clipping
Range clipping	LiDAR latency
LiDAR latency	Control latency
Control latency	Dropout
Normal	Normal

Note: The normal label was unchanged. Standard and label-deranged routing were executed in separate closed-loop rollouts with paired environment and fault seeds; identical trajectories, diagnosis sequences, or switching sequences were not assumed.

Table S6. Evaluation Episode Accounting

Experiment block	Scope	Records
Central evaluation	Five methods across core, severity, and dynamic-transition suites	160,500
Extended core baselines	Robust-100k, robust-200k, and temporal-robust-200k	31,500
Extended severity baselines	Three robust baselines across 18 fault-severity conditions	32,400
Seven-method dynamic block	Seven methods, 18 scenarios, and 200 episodes per scenario and seed	75,600
Mixed/unknown stress block	Five methods, 14 condition-severity cells, and 200 episodes per cell and seed	42,000
Router-component ablations	Five ablations, 18 scenarios, and 100 episodes per scenario and seed	27,000
Transition-time sensitivity	Steps 20 and 100, five methods, and 18 scenarios	54,000
Interaction-matched baseline	Core, severity, transition, mixed/unknown, and correlated protocols	42,900
Fault-policy matrix	Normal, robust, and six specialist policies	60,000
Dedicated route tracing	Diagnosed routing under seven mixed/unknown conditions at two severities	4,200
Label-derangement control	Two routing conditions across 18 transition scenarios	10,800
Additional correlated controls	Robust-100k, robust-200k, temporal-robust-200k, and diagnosed switching	9,600
Complete evaluation record	All separately executed blocks	**550,500**

Note: Separately executed repeated method-condition cells were counted as distinct episode records but not as additional policy-training replications. The 9600-record correlated-control block excluded the interaction-matched robust policy. Its 2400 correlated-fault records were counted only within the 42,900-record interaction-matched family.

Table S7. All-Severity Specialist Cross-Performance Summary

Policy grouping	Success rate
Fault-matched specialists	0.934 ± 0.007
Non-corresponding specialists	0.510 ± 0.016
Robust-100k	0.872 ± 0.012

Note: Results aggregate the six injected fault modes at severities 0.15, 0.35, 0.55, and 0.75. For each policy-training seed, the fault-matched value was computed from the diagonal fault–specialist combinations, the mismatched value from all off-diagonal specialist combinations, and the robust-100k value from the corresponding robust-policy conditions. Entries report mean ± sample SD across the three policy-training seeds.
